# Analyses of genome wide association data, cytokines, and gene expression in African-Americans with benign ethnic neutropenia

**DOI:** 10.1371/journal.pone.0194400

**Published:** 2018-03-29

**Authors:** Bashira A. Charles, Matthew M. Hsieh, Adebowale A. Adeyemo, Daniel Shriner, Edward Ramos, Kyung Chin, Kshitij Srivastava, Neil A. Zakai, Mary Cushman, Leslie A. McClure, Virginia Howard, Willy A. Flegel, Charles N. Rotimi, Griffin P. Rodgers

**Affiliations:** 1 Center for Research on Genomics and Global Health, National Human Genome Research Institute, National Institutes of Health (NIH), Bethesda, Maryland, United States of America; 2 Molecular and Clinical Hematology Branch, National Institute of Diabetes and Digestive and Kidney Diseases, NIH, Bethesda, Maryland, United States of America; 3 National Institute of Biomedical Imaging and Bioengineering, NIH, Bethesda, Maryland, United States of America; 4 Warren Grant Magnuson Clinical Center, NIH, Bethesda, Maryland, United States of America; 5 Departments of Pathology and Medicine, University of Vermont Larner College of Medicine, Burlington, Vermont, United States of America; 6 School of Public Health, University of Alabama, Birmingham, Alabama, United States of America; 7 Department of Epidemiology and Biostatistics, Drexel University, Philadelphia, Pennsylvania, United States of America; University of Texas Health Science Center at San Antonio, UNITED STATES

## Abstract

**Background:**

Benign ethnic neutropenia (BEN) is a hematologic condition associated with people of African ancestry and specific Middle Eastern ethnic groups. Prior genetic association studies in large population showed that rs2814778 in Duffy Antigen Receptor for Chemokines (*DARC*) gene, specifically DARC null red cell phenotype, was associated with BEN. However, the mechanism of this red cell phenotype leading to low white cell count remained elusive.

**Methods:**

We conducted an extreme phenotype design genome-wide association study (GWAS), analyzed ~16 million single nucleotide polymorphisms (SNP) in 1,178 African-Americans individuals from the Reasons for Geographic and Racial Differences in Stroke (REGARDS) study and replicated from 819 African-American participants in the Atherosclerosis Risk in Communities (ARIC) study. Conditional analyses on rs2814778 were performed to identify additional association signals on chromosome 1q22. In a separate cohort of healthy individuals with and without BEN, whole genome gene expression from peripheral blood neutrophils were analyzed for DARC.

**Results:**

We confirmed that rs2814778 in *DARC* was associated with BEN (*p* = 4.09×10^−53^). Conditioning on rs2814778 abolished other significant chromosome 1 associations. Inflammatory cytokines (IL-2, 6, and 10) in participants in the Howard University Family Study (HUFS) and Multi-Ethnic Study in Atherosclerosis (MESA) showed similar levels in individuals homozygous for the rs2814778 allele compared to others, indicating cytokine sink hypothesis played a minor role in leukocyte homeostasis. Gene expression in neutrophils of individuals with and without BEN was also similar except for low DARC expression in BEN, suggesting normal function. BEN neutrophils had slightly activated profiles in leukocyte migration and hematopoietic stem cell mobilization pathways (expression fold change <2).

**Conclusions:**

These results in humans support the notion of DARC null erythroid progenitors preferentially differentiating to myeloid cells, leading to activated DARC null neutrophils egressing from circulation to the spleen, and causing relative neutropenia. Collectively, these human data sufficiently explained the mechanism DARC null red cell phenotype causing BEN and further provided a biologic basis that BEN is clinically benign.

## Introduction

Total and differential white blood cell (WBC) counts are often used as measures of health, immunocompetence, and tolerance to chemotherapy with neutrophils usually accounting for 40% to 80% of total WBC. Asymptomatic or benign reductions in neutrophils (absolute neutrophil count, or ANC, <1.5×10^9^ cells/L) are primarily associated with non-white ethnicity, hence the term benign ethnic neutropenia (BEN). BEN has been documented in individuals with ancestry from Yemen, the Middle East (Bedouin Arabs), Africa (including admixed African populations in the Americas) and Europe [1–5]. The largest population-based study of neutropenia in the United States (US) showed ethnic differences in BEN prevalence, with estimates of 4.5%, 0.79% and 0.38% among African-Americans, European-Americans, and Mexican-Americans, respectively [4].

There is substantial evidence for the genetic control of hematologic traits in general and BEN in particular. First, hematologic traits (including WBC and neutrophil counts) showed high heritability, with estimates of 61% to 96% in twin studies [6, 7] and 42% to 62% in other kinds of studies [8]. Second, admixture mapping studies identified a chromosome 1q22 polymorphism within the *DARC* gene that strongly influenced WBC counts in persons of African ancestry. Indeed, *DARC* (also known as *ACKR1*, atypical chemokine receptor 1—Duffy blood group) explained up to 20% of the variance in WBC and neutrophil counts [8–10]. Third, GWAS have also identified genetic loci associated with hematologic traits, including WBC and neutrophil count. However, no GWAS for BEN has been performed to date.

In the present study, we conducted a GWAS of WBC in African-Americans using an extreme phenotype design. We reasoned that a study in African-Americans may provide fresh insight into the genetics of BEN, especially when genome-wide dense SNP array analysis is combined with gene expression studies.

## Methods

The study protocol was approved by the Institutional Review Board (IRB) of National Human Genome Research Institute (NHGRI) and National Heart, Lung, and Blood Institute (NHLBI) at the National Institutes of Health. The use of the Atherosclerosis Risk in Communities study (ARIC) replication sample was approved by the NHGRI IRB and the National Center for Biotechnology Information (NCBI) database of Genotypes and Phenotypes (dbGaP) Data Access Committee (DAC). All study participants gave written informed consent prior to inclusion in the studies. Each study complies with the tenets of the Declaration of Helsinki.

### Subjects for discovery GWAS

The REGARDS cohort comprised 30,239 self-identified African-American and white individuals, aged 45 and older at enrollment in 2003–2007. Fifty-six percent of the sample was from the eight southeastern United States comprising the ‘stroke belt’ with the remainder from the other 40 contiguous states; 42% are African-American, and 55% women [11]. A baseline telephone interview assessed cardiovascular risk factors (including smoking history). An in-home physical assessment conducted 3–4 weeks after the telephone interview obtained blood samples.

The subset selected for this BEN discovery GWAS were self-identified African-Americans. Variation in the neutrophil count was directly reflected in WBC or total leukocyte count. Therefore, WBC, a parameter in the complete blood count testing, was a very good surrogate marker for ANC. An extreme phenotype study design was used, with 600 participants with WBC counts in the 1^st^–8^th^ percentile at one extreme (low WBC or “LW”) and 600 participants with counts in the 75^th^–99^th^ percentile at the other extreme (high WBC or “HW”). Participants with WBC below 1^st^ or over 99^th^ percentile were excluded to reduce the influence of pathologic alterations in WBC counts.

### Genotyping and quality control

Genomic DNA was extracted from banked WBC using the Puregene DNA purification kit. DNA quantification was conducted using the PicoGreen method. Genotyping was conducted at the Center for Inherited Disease Research (CIDR) on 1,260 REGARDS samples (including technical replicates and related samples) using the Illumina HumanOmniExpress-12v1 array. A total of 1,247 (99.0%) of the attempted 1,260 samples were successfully assayed, representing 1,178 unrelated individuals after exclusion of technical replicates and cryptic relatives (kinship coefficient > 0.125). After application of technical filters, CIDR released 730,525 SNPs. Characteristics of the subjects in the discovery GWAS are shown in Table A in S1 File.

Stringent quality control (QC) measures were instituted at various stages of the project. First, QC samples, including 26 HapMap samples, as well as related individuals and duplicate samples, were genotyped along with the LW GWAS samples. This facilitated identification of poorly performing SNPs showing duplicate inconsistencies and/or Mendelian inconsistencies. Second, the resulting genotypes were passed through a set of filters, including departures from Hardy-Weinberg equilibrium, locus missingness, sample missingness, minor allele frequency, sex differences in allelic frequency, extreme heterozygosity (for autosomal SNPs), non-observance of founder genotypes, and observance of heterozygous haploid genotypes. Regions with large chromosomal anomalies were filtered out. A total of 22,327 SNPs of the original 730,525 SNPs were filtered out and 656,747 SNPs were carried forward for analysis. Third, population genetic characteristics of the study sample were assessed by computing principal components (PCs) of the genotypes and evaluating cluster patterns for outliers. Study sample PCs were also computed and compared to the HapMap population reference panels to verify ancestry. The study sample is African-American, an admixed African-European group. Therefore, we expect the genotypes of our sample to cluster on a continuum between West African and European reference genotypes. Fourth, an evaluation of the sample for potential population stratification was conducted as described in the next section.

### Population structure

We evaluated clustering of the samples and other characteristics associated with population structure by computing the PCs of the genotyped SNPs. SNPs for this analysis were obtained by linkage disequilibrium-based pruning of the dataset (parameters: variance inflation factor (VIF) of 1.1 for a 50 SNP window with a slide of 5 SNPs). The VIF is equivalent to 1/(1-R^2^), in which R^2^ represents the multiple correlation coefficient for a SNP regressed on all other SNPs in the window. Using this process, 94,360 SNPs were extracted for assessment of population structure in the REGARDS sample. We evaluated the number of significant PCs using the Minimum Partial Average (MAP) Test [12]. This test is known to perform better than the Tracy-Widom test in identifying the number of significant principal components in admixed populations. As shown in Figure A in S1 File, the REGARDS participants clustered tightly together as a single cluster with no significant outliers. Projection of the REGARDS participants on to four HapMap/1000 Genomes samples (YRI, CEU, JPT, and CHB) demonstrated a distribution along a line spanning between the African (YRI) and European (CEU) reference samples (Figure B in S1 File), a pattern consistent with African-European admixture. Only the first PC explained a significant proportion of variance of the genotypes (Figure C in S1 File), confirmed by the MAP test. Therefore, the first PC was included as a covariate in the association analyses.

### Imputation

To improve genomic coverage, *in silico* imputation was conducted using the 1000 Genomes reference data (http://www.1000genomes.org). Imputation of non-genotyped SNPs was conducted at the University of Washington, Genetics Coordinating Center (GCC) using IMPUTE2 (https://mathgen.stats.ox.ac.uk/impute/impute_v2.html), following pre-phasing with SHAPEIT2 version 2 (https://mathgen.stats.ox.ac.uk/genetics_software/shapeit/shapeit.html). The 1000 Genomes Project’s worldwide reference panel Phase 1 Version 3 (ftp://ftp-trace.ncbi.nih.gov/1000genomes/ftp/phase1/) comprising all 11 samples was used to impute non-genotyped SNPs. Inclusion in imputation processing required there be at least two copies of the minor allele in the African or European samples. Additionally, only SNPs were imputed (approximately 1.5 million indels and structural variants were excluded from imputation). Imputed SNPs with an “info” score <0.3 were considered low quality and filtered out from the dataset.

### Association analysis

Logistic regression association analysis was performed under an additive genetic model with the imputed dosages using PLINK v1.07 (http://pngu.mgh.harvard.edu/~purcell/plink/), including age, sex, smoking status, and the first PCs as covariates in the model. Annotation of significant hits was conducted using the R package NCBI2R (http://cran.r-project.org/web/packages/NCBI2R/NCBI2R.pdf). *In silico* lookup of previously reported genome-wide significant SNPs with WBC and/or neutrophil counts was done with the aid of the Catalog of Published Genome Wide Association Studies (http://www.ebi.ac.uk/gwas).

### Replication in the Atherosclerosis Risk in Communities (ARIC) study

Replication was conducted using African-American participants from ARIC. The phenotype definitions were the same as in the discovery GWAS. Characteristics of the study subjects are shown in Table B in S1 File. These samples were genotyped on the Affymetrix Genome-Wide Human SNP Array 6.0 and imputed into the 1000 Genomes phase 1 version 3 reference panel. Details regarding genotyping, data cleaning, imputation and analyses are available in dbGaP and mirror those implemented in the discovery GWAS in the REGARDS study as described above. Phenotypes and genotypes (including imputed genotypes) were obtained from dbGAP under controlled access. Association analysis was performed under an additive genetic model, using the same covariates as in the discovery GWAS.

### Gene expression analysis

Participants for the gene expression study were enrolled at the NIH Clinical Center, Bethesda, Maryland, protocol 03-H-0168 (clinicaltrials.gov, NCT00059423). This study consisted of seven individuals with absolute neutrophil count <1.5x10^9^ cells/L and five non-BEN individuals with absolute neutrophil count >4.0x10^9^ cells/L. All subjects were self-identified African-Americans and nonsmokers. Demographic characteristics of the subjects are shown in Table C in S1 File. Whole blood was centrifuged using density gradient separation (Ficoll) to obtain granulocytes. Approximately 10^7^ granulocytes were mixed with 1 mL RNA Stat-60 solution. RNA was extracted using chloroform, precipitated with isopropanol, and rehydrated with DEPC-treated water.

Affymetrix Human Gene 2.0 ST arrays were used to evaluate gene and exon expression. This array has ~48,000 gene-level probe sets and ~418,000 exon-level probe sets. The array has multiple probes for each exon of each transcript and has a median of 21 unique probes for each transcript. This design permits the analysis of expression at both the gene and exon levels and facilitates the study of transcript variants and alternative splicing events. Array processing, including hybridization, scanning and washing, was done following the manufacturer’s instructions. Data were deposited in NCBI Gene Expression Omnibus (GEO), series record GSE108894, https://www.ncbi.nlm.nih.gov/geo/query/acc.cgi?acc=GSE108894. Data quality control and analysis were done using Partek Genomics 6.6 (Partek, Inc., St. Louis, MO). Affymetrix CEL files were imported into Partek, followed by probe summarization and normalization using the RMA (Robust Muti-Chip Average) algorithm. Based on PC analysis, one outlier was removed from further analysis. Probe sets with low expression (log_2_ value < 3.0) in all samples were excluded.

Gene-level differential expression between BEN and non-BEN was assessed by analysis of covariance, controlling for the effects of age, sex, and batch after mean summarization of exon-level probes to genes. Exon-level differential expression between BEN and non-BEN was assessed by analysis of covariance, controlling for the effects of age, sex, and batch. For both gene-level and exon-level analysis, we considered a false discovery rate (FDR) < 0.05 as significant. The Partek alternative splicing workflow ANOVA model was used to detect alternatively spliced variants, adjusting for age, sex, and batch. Since there is no consensus in the field on how to appropriately correct for multiple comparisons in alternative splicing, we considered two thresholds: an unadjusted *p*-value <0.05 and an FDR <0.05. To declare an alternative splicing event, we filtered for genes showing differential expression between BEN and non-BEN.

We used Thomson Reuters’ GeneGo MetaCore™ (https://portal.genego.com/) to identify pathways represented greater than expected by chance from gene lists of interest, *i*.*e*., differentially expressed and/or alternatively spliced transcripts. MetaCore is based on a high-quality, manually-curated database of transcription factors, receptors, ligands, kinases, drugs, and endogenous metabolites as well as species-specific directional interactions between protein-protein, protein-DNA, and protein-RNA, drug targeting, and bioactive molecules and their effects. We used MetaCore to search for canonical networks for which our gene sets of interest are enriched.

### DARC gene expression

Total neutrophil RNA from participants enrolled at the NIH Clinical Center was transcribed to cDNA (SuperScript III First–Strand Synthesis SuperMix; Invitrogen, Carlsbad, CA). Quantitative real-time PCR was performed (SYBR Green chemistry, CFX96 Real-Time PCR Detection System; Bio-Rad, Hercules, CA, USA) to assay the two known *DARC* gene transcripts (NM_001122951.2, short transcript of 1258 bp and NM_002036.3, long transcript of 2024 bp). The relative expression of the transcripts was calculated using the comparative C_T_ (2^−ΔΔC^_T_) method [13] with *GAPDH* as an internal control gene for normalization. Samples with *GAPDH* expression of ≤ 23 C_T_ were included in the analysis. A Caucasian sample with the Fy(a+b+) phenotype was used as calibrator and set at 100%. All samples were tested in triplicate, and the assay was performed three times.

### Cytokine analyses

Trait values for cytokines previously reported to be associated with DARC or Duffy expression–namely C-reactive protein (CRP), interleukin 2 (IL-2), IL-6, IL-10, matrix metallopeptidase 3 (MMP-3) and 9 were retrieved from participants in the Howard University Family Study (HUFS, N = 1623) and Multi-Ethnic Study in Atherosclerosis (MESA, N = 1344). CRP, IL-6, and IL-10 were available from HUFS; CRP, IL-2, IL-6, MMP-3, and MMP-9 in MESA. Cytokine values were log-transformed under a recessive model and analysis adjusted for age, sex, body mass index and type 2 diabetes.

## Results

### Discovery GWAS in REGARDS

The discovery GWAS included 592 LW and 586 HW individuals (Table A in S1 File). Both groups were similar in age but differed in the prevalence of smoking and CRP levels. The mean individual admixture proportion was 80.7% African ancestry. Individual admixture proportion was significantly correlated with LW, with risk increasing with increasing proportion of African ancestry (odds ratio, OR = 12.6, *p* = 9.84×10^−7^).

**Fig 1:**
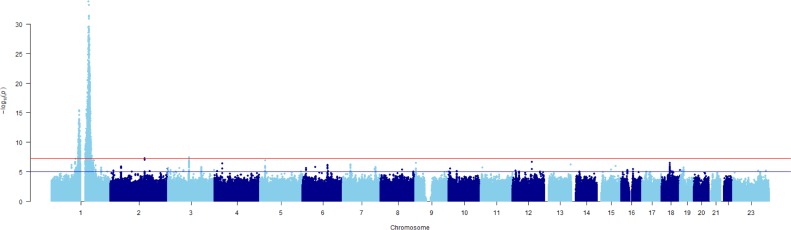
Manhattan plot for discovery GWAS for BEN: The REGARDS study.

**Fig 2:**
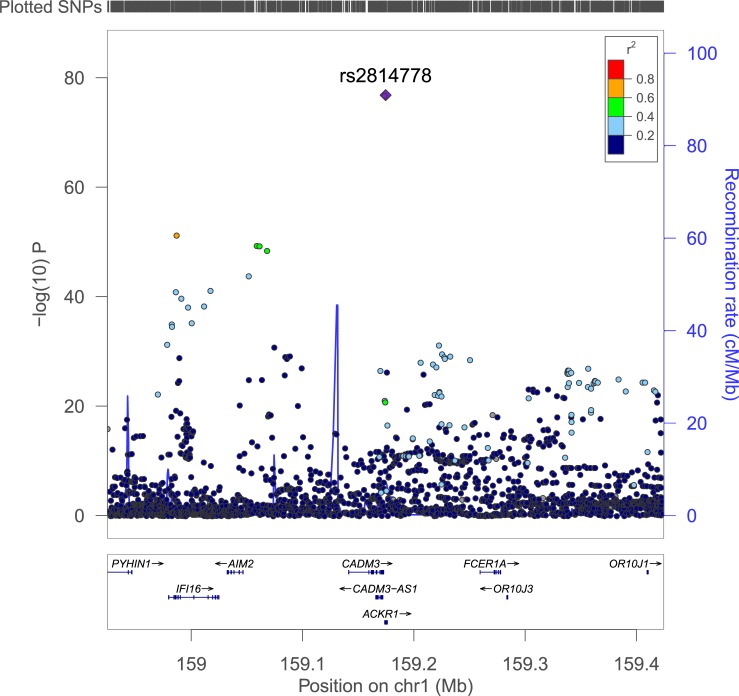
Regional association plot for chromosome 1 genome wide significant locus centered on *DARC*.

The Manhattan plot for the association analysis is shown in Fig 1. The top locus was a broad region on chromosome 1, with the leading SNP being *DARC* SNP rs2814778 (OR = 0.0641, *p* = 4.09×10^−53^). This was the SNP that has been most frequently reported in previous studies. Notably, the two top hits in *DARC* (SNPs rs2814778 and rs12075) defined the Duffy blood group system and its two principal antigens (Fy^a^ and Fy^b^) of the Duffy blood group system. The top non-*DARC* significant SNP was rs856046, an intronic variant located in the gene interferon gamma-inducible protein 16 (*IFI16*) (OR = 9.6175, *p* = 2.89×10^−40^). The leading variants associated with LW and their genic annotation are listed in Table 1. A regional plot around the index SNP is shown in Fig 2 and an annotation of the genes in this region is shown in Table D in S1 File. The only genome-wide significant hit outside of chromosome 1 was rs36076607 (OR = 1.61, *p* = 4.67×10^−8^) in the ephrin receptor A3 (*EPHA3*) gene.

**Table 1:** Most significant loci in discovery GWAS.

SNP	CHR	BP	A1	FRQ	OR	SE	P	SNP class	Gene
rs2814778	1	159174683	T	0.208	0.064	0.179	4.09E-53	upstream-2KB, utr-5-prime	*DARC*
rs856046	1	158987941	A	0.829	9.617	0.170	2.89E-40	intron	*IFI16*
rs11577338	1	155240077	G	0.850	5.549	0.161	2.66E-26	intron	*CLK2*
rs6696888	1	155508882	G	0.8535	5.276	0.159	2.32E-25	intron	*ASH1L*
rs4074436	1	154531910	G	0.731	3.120	0.112	5.84E-24	upstream-2KB	*UBE2Q1*
rs670523	1	155878732	A	0.825	4.331	0.145	7.45E-24	intron	*RIT1*
rs863000	1	159170986	T	0.875	6.525	0.188	2.77E-23	nc-transcript, utr-3-prime	*LOC100131825*
rs2501339	1	159825061	G	0.132	0.190	0.170	2.74E-22	intron,utr-5-prime	*C1orf204*
rs10908720	1	159408803	C	0.824	3.698	0.135	4.54E-22	upstream-2KB	*OR10J1*
rs6699071	1	157494812	A	0.616	2.459	0.098	4.33E-20	intron	*FCRL5*
rs12723848	1	162237979	G	0.868	4.267	0.158	4.79E-20	intron	*NOS1AP*
rs4845700	1	154981708	C	0.681	2.542	0.101	5.33E-20	intron	*ZBTB7B*
rs7516146	1	159801111	G	0.893	5.443	0.186	1.10E-19	intron	*SLAMF8*
rs855871	1	159044651	G	0.611	2.458	0.099	1.19E-19	intron	*AIM2*
rs12025136	1	161980502	C	0.787	2.945	0.119	1.69E-19	intron	*OLFML2B*
rs6682716	1	156551848	A	0.810	3.205	0.129	1.79E-19	missense	*TTC24*
rs411088	1	158171295	C	0.633	2.424	0.098	3.55E-19	intron	*LOC100505799*
rs2789424	1	159942456	C	0.728	2.615	0.107	5.26E-19	intron	*LOC100505633*
rs1065457	1	158324425	A	0.188	0.326	0.127	1.71E-18	intron, missense	*CD1E*
rs11576266	1	156572159	A	0.802	2.965	0.125	4.07E-18	upstream-2KB	*GPATCH4*
rs4845490	1	152849299	A	0.359	0.423	0.099	4.80E-18	upstream-2KB	*SMCP*
rs1704745	1	162693795	A	0.853	3.647	0.150	7.15E-18	intron	*DDR2*
rs1633276	1	158903815	C	0.418	2.914	0.124	1.02E-17	intron	*PYHIN1*
rs942679	1	156351066	C	0.885	4.32	0.171	1.57E-17	intron	*RHBG*
rs7529060	1	157725264	T	0.140	0.277	0.150	1.61E-17	intron	*FCRL2*
rs539118	1	150966197	G	0.708	2.526	0.108	1.69E-17	intron	*ANXA9*
rs2249707	1	159922959	C	0.688	2.458	0.105	1.71E-17	intron	*SLAMF9*
rs12401997	1	156147796	C	0.909	5.824	0.208	2.45E-17	downstream-500B	*SEMA4A*
rs1320489	1	157977491	C	0.92	9.207	0.262	2.92E-17	intron	*KIRREL*
rs505058	1	156106185	T	0.393	0.45	0.094	3.00E-17	nc-transcript, synonymous-codon	*LMNA*

Top 30 genome-wide significant genes in chromosome 1 region by leading SNP

#### Conditional analyses on the leading *DARC* variant

Given that the association on chromosome 1 encompasses a broad region of natural selection in African-ancestry populations (Figure D in S1 File), *post hoc* analyses were conducted to condition on the leading variant in *DARC* (rs2814778). Finding genome-wide significant signal in this analysis would help resolve the issue of whether there are other loci in this region apart from *DARC* that influence risk of low WBC. Therefore, the GWAS was repeated with conditioning on rs2814778. The results of the conditional analysis showed no other genome-wide significant signal in the chromosome 1 region. There were also no other genome-wide significant SNPs across the genome; the best SNP was rs77998448 (*p* = 1.4×10^−7^, Table E in S1 File).

#### Analysis of *DARC* SNPs, rs12075 and rs2814778

Two SNPs defined the DARC phenotype. Rs12075*A (FY*B) is ancestral, from which rs12075*G (FY*A) is derived. FY*01N.*01*, or *DARC* null, is independently derived from FY*B and is defined by rs2814778, with the ancestral allele *T* and the derived allele *C*. The ancestral haplotype carried allele *A* at rs12075 with allele *T* at rs2814778; two derived haplotypes were *G* with *T* (FY*A) and *A* with *C* (FY*01N.*01*), respectively. The predicted DARC phenotype frequency computed from the allele frequencies is shown in Table F in S1 File. As expected, the predicted frequencies of Fy(a+b+), Fy(a-b+), and Fy(a+b-) phenotypes were all higher in HW, whereas the Fy(a-b-) phenotype was predominantly found in LW. Interestingly, 37% of the HW group also carried the Fy(a-b-) phenotype.

#### Replication analysis in ARIC

Replication analysis was performed in ARIC using the same methods and models as in the discovery GWAS. Characteristics of the ARIC participants are shown in Table B in S1 File. Association analysis showed a similar chromosome 1 peak, with the same leading variant: *DARC* SNP rs2814778 (*p* = 2.84×10^−21^) and with the same direction of effect. The second leading variant in the replication study was rs2570916 (OR = 10.7, *p* = 3.11×10^−18^), an *IFI16* intronic variant. Most of the genome-wide significant chromosome 1 variants identified in the discovery sample were replicated in the ARIC sample but at different levels of significance (Table G in S1 File). However, the only non-chromosome 1 genome-wide significant hit in the discovery study—rs36076607 (*EPHA3*)*—*was not replicated in the ARIC sample (*p* = 0.871, Table H in S1 File).

#### In silico lookup of previous GWAS findings for WBC and/or neutrophil counts

We extracted from the GWAS Catalog (accessed 7/10/2014) genome-wide significant SNPs that were associated with WBC and/or neutrophil counts. The SNPs that were significant in the present study are shown in Table 2.

**Table 2:** *In silico* replication of previously reported GWAS-significant WBC-related traits.

SNP	CHR	Gene	Reference	Trait*	A1	FRQ	OR	SE	P
rs2814778	1	*DARC*	Crosslin et al 2011	WBC (AA)	T	0.208	0.064	0.179	4.09E-53
			Ramsuran et al 2011	Neutrophil count					
rs12075	1	*DARC*	Crosslin et al 2011	WBC (AA)	G	0.082	0.137	0.228	4.21E-18
rs2837828	21	*DSCAM*	Ramsuran et al 2011	Neutrophil count	A	0.782	0.750	0.107	7.41E-3
rs454305	2	*TGFA*	Ramsuran et al 2011	Neutrophil count	A	0.352	1.246	0.091	1.62E-2
rs11968166	6	*CCND3*	Ramsuran et al 2011	Neutrophil count	G	0.889	1.341	0.136	3.14E-2
rs4422476	4	[Intergenic]	Ramsuran et al 2011	Neutrophil count	T	0.215	0.821	0.107	6.62E-2
rs1371799	4	[Intergenic]	Reiner et al 2011	WBC	T	0.764	0.831	0.102	7.11E-2
rs12772794	10	[Intergenic]	Ramsuran et al 2011	Neutrophil count	A	0.869	0.789	0.132	7.32E-2
rs445	7	*CDK6*	Okada et al 2011	WBC types (Neutrophil count)	C	0.803	0.832	0.106	8.67E-2
			Reiner et al 2011	WBC					
rs3859192	17	*GSDMA*	Crosslin et al 2011	WBC (EA)	C	0.661	1.163	0.089	9.03E-2

### Gene expression analysis

**Fig 3:**
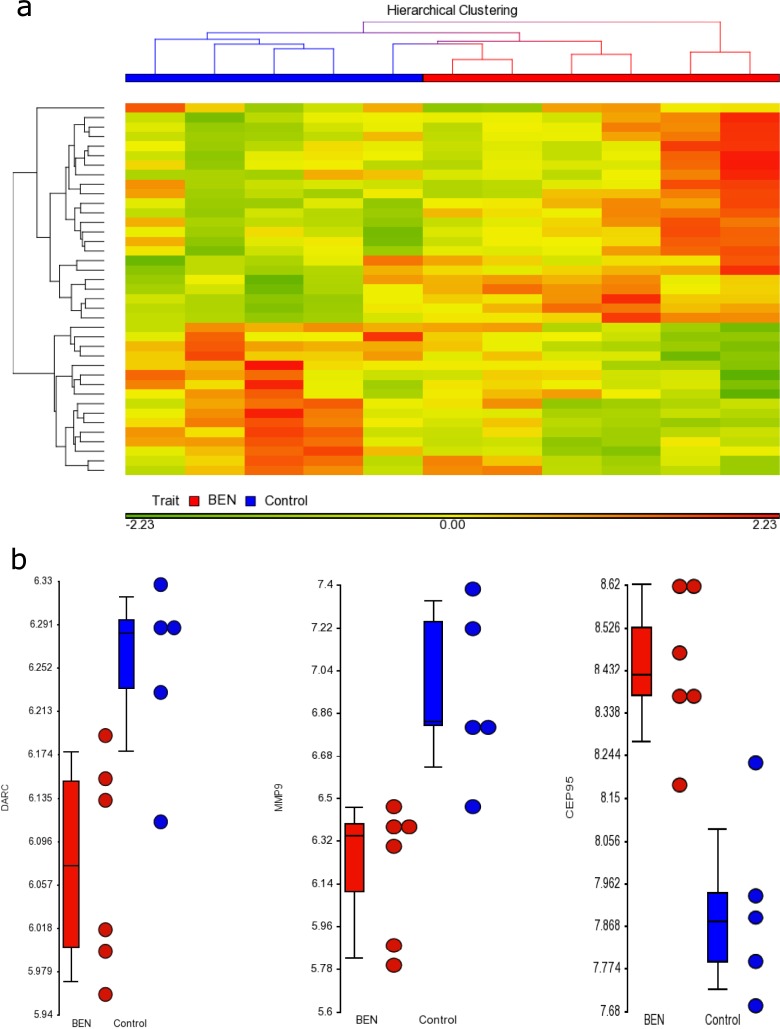
Granulocyte gene expression. A. Heat plot of most differentially expressed transcripts in granulocytes of BEN compared to non-BEN individuals B. Dot plot of gene expression of DARC (the gene most significant on GWAS) and MMP9 (the gene with the largest absolute differential expression between BEN and non-BEN individuals).

For gene-level expression, no transcript showed significantly different expression between BEN and non-BEN samples at an FDR <0.05. This finding suggested that neutrophils from BEN individuals would be functionally similar to neutrophils from non-BEN individuals. Based on nominal p-values, the most significantly differentiated genes between the two groups (Fig 3) included *CRX* (*p* = 1.04×10^−6^, fold change BEN cases relative to controls -1.35), *LCP1* (*p* = 6.54×10^−5^, fold change -1.27), *CEP95* (*p* = 8.30×10^−5^, fold change 1.65), *HECTD4* (*p* = 9.37×10^−5^, fold change 1.14), and *RGS20* (*p* = 9.59×10^−5^, fold change 1.21). The list of genes with unadjusted *p*<5×10^−4^ (absolute fold change between BEN cases and controls of 1.2–1.8) is shown in Table I in S1 File. Enrichment analyses of this gene set in hematologic and immune systems showed that the top scoring canonical pathway maps were: Development_EGFR signaling pathway (p = 0.009), Development_Role of proteases in hematopoietic stem cell mobilization (p = 0.041), Development_Role of G-CSF in hematopoietic stem cell mobilization (p = 0.047), and Development_Role of HGF in hematopoietic stem cell mobilization (*p* = 0.047, Table J and Figure P in S1 File). Repeating this analysis in all tissues showed that these four pathway maps remained among the top six scoring pathway maps (Table K in S1 File). Gene ontology (GO) enrichment scoring of hematologic and immune system processes showed that leukocyte migration had the highest enrichment score (ES 3.69, Figure Q in S1 File). For exon-level expression, no exon was differentially expressed at an FDR of 0.05 or at a Bonferroni-adjusted *p*-value of 1.42×10^−7^ (Table L in S1 File).

An analysis of differential alternative splicing in BEN compared to non-BEN showed that, at an unadjusted *p*-value of 0.05, 1,945 genes had a high probability of differential alternative splicing. Adjusting for multiple comparisons using the FDR, 16 genes were significant at an FDR <0.05 (Table M in S1 File). A total of 56 genes were alternatively spliced but not differentially expressed (alternative splicing *p*-value < 0.05, differential expression *p*-value >0.95, Table N in S1 File).

**Fig 4:**
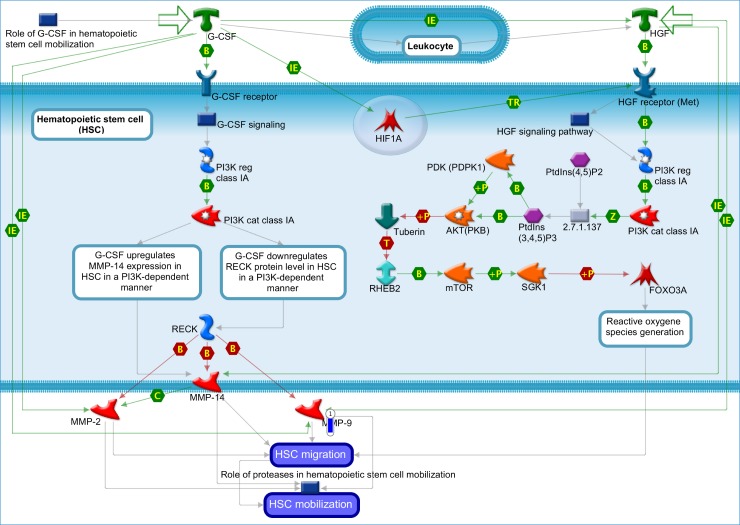
Canonical pathways under-expressed in BEN compared to non-BEN individuals.

We sought to identify differentially expressed transcripts that were coded for by genes within the chromosome 1 genome-wide significant association region. At *p*<0.05, there were seven such transcripts, including *DARC* (Fig 4, Table 3). Therefore, the locus that displayed the greatest evidence for statistical genetic association with BEN also showed differential expression between BEN and non-BEN controls. We then confirmed with real-time PCR that *DARC* expression was indeed absent in the same BEN individuals as in the gene expression cohort (Table 4).

**Table 3:** Most differentially expressed genes in GWAS-significant chromosome 1 region.

Transcript ID	Gene Symbol	RefSeq	*p*-value (BEN *vs*. Control)	Fold-change (BEN *vs*. Control)
**Genome-wide analysis**				
16863691	*CRX*	ENST00000221996	1.04E-06	-1.35
16778849	*LCP1*	NM_002298	6.54E-05	-1.27
16837065	*CEP95*	ENST00000556440	8.30E-05	1.65
16770344	*HECTD4*	NM_001109662	9.37E-05	1.14
17068938	*RGS20*	NM_170587	9.59E-05	-1.22
16914395	*MMP9*	NM_004994	1.35–04	-1.81
16898788	*TGFA*	NM_003236	2.00–04	-1.40
16855684	*KDSR*	ENST00000406396	2.21–04	1.60
16860302	*ZNF91*	ENST00000595893	2.61–04	1.38
16771146	*GCN1L1*	ENST00000300648	2.75–04	1.38
16738174	*FNBP4*	NM_015308	2.84–04	1.46
16664708	*ZFYVE9*	NM_004799	2.92–04	1.53
16928938	*SEC14L2*	NM_033382	3.16–04	1.48
**GWAS- significant chromosome 1 region**				
16694556	*C1orf61*	ENST00000368243	0.0124	-1.18
16694135	*CLK2*	NM_003993	0.0172	1.33
16694661	*GPATCH4*	ENST00000368232	0.0176	1.64
16693924	*DCST2*	NM_144622	0.0196	-1.26
16694472	*SMG5*	NM_015327	0.0249	1.27
16694728	*MRPL24*	NM_145729	0.0338	1.30
16672428	*DARC*	NM_002036	0.0395	-1.16
16695151	*CRP*	NM_000567	0.0583	-1.17

**Table 4:** Relative expression of *DARC (ACKR1)* gene transcripts.

Sample	Ethnicity	Duffy phenotype	*ACKR1* transcript expression [mean ± SD (%)] *			
			Short	Long	Both	n
Low WBC	African-American	Fy(a-b-)	0 ± 0	0 ± 0	0 ± 0	4
High WBC	African-American	Fy(a-b-)	0 ± 0	0 ± 0	0 ± 0	2
High WBC	African-American	Fy(a+b-)	74.7 ± 48.7	8.7 ± 0.03	68.5 ± 32.6	1
Calibrator	Caucasian	Fy(a+b+)	100	100	100	1

* The assay shown was replicated twice with comparable results.

### Cytokine studies

Given the postulated role of DARC as a ‘cytokine sink’, which binds to inflammatory cytokines and attracts leukocytes/neutrophils into peripheral blood, we tested the DARC rs2814778 *C* allele in the homozygote state (i.e. *DARC* null) for association with cytokines in two cohorts: HUFS and MESA. The findings showed that rs2814778 *CC* was significantly associated with higher CRP levels in both HUFS (β = 0.099 (SE 0.026), p < 0.0001) and MESA (β = 0.148 (SE 0.061), p = 0.015) (Table O in S1 File). While the direction of effect was also positive for IL-2, IL-6 and IL-10, the p-values were not significant. Additionally, we also tested for metalloproteases that are commonly associated with neutrophil function. MMP-9 was associated with the Duffy null state with a negative direction of effect—β = -0.439 (SE 0.084), p < 0.001 (Table O in S1 File). This finding is consistent with our gene expression analysis which showed that MMP-9 was significantly down-regulated in BEN cases when compared with controls (Table 3, Fig 3). IL-8 and MCP1 were not available for analyses in HUFS or MESA cohorts.

## Discussion

We report a genome-wide analysis for LW (BEN) that combined a GWAS approach and whole genome gene expression analysis in African-Americans. BEN is disproportionately observed in specific ethnic groups, including people with African or Middle East ancestry. While BEN is not associated with infection, immunodeficiency, or any other clinical pathology, BEN may have clinical implications for chemotherapy, immunosuppressant therapy, organ transplantation, definitions for clinical conditions such as sepsis, and treatment with psychotropic medications [14, 15]. Specifically, if the etiology of LW (BEN) was benign, this would provide reassurance to continue treatment. If the etiology was otherwise, withholding treatment would be logical. Our strongest association was on chromosome 1 and centered on *DARC*, a similar finding to the association reported in GWAS of WBC and/or neutrophil count in African-Americans [16, 17].

However, WBC and neutrophil counts in the general population are influenced by both genetic and non-genetic factors (such as age, smoking, and use of specific medications). Loci on chromosomes 6, 12, 17, and 20 have been associated with WBC in European and/or Japanese populations, while loci on chromosome 1 (*DARC*), 4, and 16 have been associated with WBC and/or neutrophil count in African-Americans (Table 4 and Table P in S1 File) [16–20]. The reported chromosome 4 locus was in the gene *CXCL2* and has been identified as being associated with WBC in Hispanic, Japanese, and European-ancestry individuals [21]. *DARC* is an example of a genetic factor that is influenced by geography and ancestry, reflecting selective pressures on the genome. The *DARC* variant rs2814778 was the top ranking variant in our sample, similar to the findings of the COGENT consortium GWAS for WBC in African-Americans [16] and other studies [3]. *DARC* resides in a region under very strong positive selection and is associated with resistance to *Plasmodium vivax*. Individuals who are homozygous for the *C* allele do not express the FYA and FYB antigens, rendering them resistant to *P vivax* malaria. This is possibly through inactivation of the Duffy binding protein II, via blocking IL-8 cleavage to the antigen which is required for the malaria parasite to enter red blood cells [22, 23]. Thus, as expected in our study cohort of all African-Americans, the Fy(a+b+) phenotype was rare. The Fy(a+b-) and Fy(a-b+) phenotypes were observed more commonly in HW, and the Fy(a-b-) phenotype overwhelmingly in LW. Surprisingly, the Fy(a-b-) phenotype was also observed in more than one-third of HW, suggesting the genetic influence on neutrophil/leukocyte count is not simply from the DARC null state.

The mechanism by which DARC affects neutrophil counts has progressed since the initial identification of *DARC*. Multiple groups have explored the cytokine sink hypothesis, which purported the presence of *DARC* was necessary to mediate blood pro-inflammatory chemokines and moderate leukocyte/neutrophil counts. Large cohort analyses of two such chemokines showed that rs12075 Asp42Gly (DARC positive) was associated with higher serum IL-8 levels [24], and higher serum monocyte chemoattractant protein 1 (MCP-1) [25, 26]. These higher blood cytokine levels partially explained the relative higher WBC counts; rs2814778 (DARC null red cells) correlated with lower chemokines levels and lower WBC counts. However, this explanation did not account for the abundant DARC expression on endothelial cells in the vasculature [27, 28]. We investigated other relevant cytokines in the cytokine sink hypothesis, and showed the levels of IL-2, 6, and 10, were similar between LW and HW individuals in the HUFS and MESA cohorts. These data suggested that this hypothesis played a minor role. Additional factors are likely present to influence WBC counts.

Recently separate investigations of DARC on HSC homeostasis and quiescence in a murine model were performed. DARC on bone marrow macrophages directly interacted with CD82 on HSCs and maintained HSC quiescence. The DARC null state, whether induced by loss of marrow macrophages through chemotherapy or by DARC knockout, led to the loss of CD82, and brought the HSCs out of quiescence into differentiation [29]. In another study, the marrow of DARC knockout mice contained more committed myeloid progenitors, and neutrophils were activated with FcRγ and CD45 by gene expression, both important in host defense [30].

However, DARC null and HSC differentiation should lead to higher numbers of leukocytes (and its subsets), which is opposite of what we currently observe in BEN. *Darc-/-* mice have similar numbers of marrow HSCs and peripheral blood leukocyte and neutrophil counts. Transplantation of bone marrow from *Darc-/-* mice into *DARC* wildtype mice recapitulated the *Darc-/-* phenotype of differentiated marrow and activated neutrophils in peripheral blood. Interestingly, activated *Darc-/-* neutrophils preferentially egressed to the spleen, leading to relative neutropenia [30]. In contrast, the reverse direction of transplantation, *Darc* wildtype marrow into *Darc-/-* mice, yielded a normal distribution and number of neutrophils. Thus, DARC null red cells, DARC null but activated neutrophils, and presence of DARC on the endothelium were all necessary for the neutropenia phenotype in steady state.

Duffy antigen has been known to be expressed primarily on red blood cells and endothelial cells, but not on neutrophils or other mature white blood cell subtypes. Our gene expression studies showed that *DARC* expression is detectable in neutrophils, and *DARC* was differentially expressed between BEN and non-BEN neutrophils. Our results added to a growing body of literature, demonstrating DARC expression on neutrophils, macrophages, and lymphocytes. For example, *DARC* was expressed at low levels in several white cell lineages, including CD19^+^ B cells, CD8^+^ T cells, CD4^+^ T cells, CD33^+^ myeloid cells, and CD14^+^ monocytes (http://biogps.org/#goto=genereport&id=2532, Figure Q in S1 File). In addition, Gene Expression Omnibus (GEO) profiles from several datasets (GDS2808, GDS2214, GDS3073, GDS2255; Figure R in S1 File) showed *DARC* expression in neutrophils at baseline and after exposure to various stimuli, as well as in case-control scenarios.

Several important themes emerged from our gene expression studies. Since gene expression in neutrophils from BEN and non-BEN individuals was similar, it is a reasonable expectation that neutrophils from BEN individuals would function similarly to neutrophils from other individuals. This finding supported the clinical observation that individuals with BEN do not have an increased incidence of infection and would mount an appropriate immunologic response to infection [31]. This provided clinically important reassurance to BEN individuals, in addition to them having normal marrow morphology. Secondly, the differentially expressed genes were in pathways related to HSC mobilization and leukocyte migration, supporting the notion that activated neutrophils egress to the spleen (and possibly other organs), leading to relative neutropenia. In our earlier report of individuals of African or Caucasian descent undergoing bone marrow harvest to donate steady state hematopoietic cells for allogeneic transplantation, we showed that the total nucleated and CD34+ cells (a marker of HSC) were lower in those of African ancestry [15]. Despite the lower number of these cells, they responded appropriately to G-CSF. Individuals of African ancestry receiving G-CSF increased WBC and neutrophil counts to higher numbers than Caucasians, and achieved similar numbers of hematopoietic progenitor cells, despite starting from lower baseline blood counts [32, 33].

In conclusion, our present work substantially advanced the understanding of BEN. The analysis of GWAS with extreme phenotype design, careful conditional analyses, and a large replication cohort confirmed that rs2814778 in *DARC* was associated with BEN. Our analysis of inflammatory cytokines, which showed similar levels in individuals homozygous for the rs2814778 *C* allele compared to others, indicated that the cytokine sink hypothesis played a minor role in leukocyte/neutrophil homeostasis. Whole genome expression profiling suggested that neutrophils in BEN individuals functioned similarly to neutrophils from non-BEN individuals. The subtle but activated BEN neutrophils in leukocyte migration and HSC mobilization pathways further supported the recent murine finding that the relative neutropenia in BEN individuals resulted from DARC null progenitors preferentially differentiating to myeloid cells, leading to activated neutrophils egressing from circulation to the spleen. Collectively, these data integrated with prior findings to explain the mechanism of DARC null red cells and neutrophils causing BEN and provided a biologic basis that BEN is clinically benign.

## Supporting information

S1 File Supporting tables and figures for the analyses of GWAS, cytokines, and gene expression of neutrophils in African-Americans with benign ethnic neutropenia. (DOCX)
