# Supplementary material for: Analyses of genome wide association data, cytokines, and gene expression in African-Americans with benign ethnic neutropenia
Source: PLoS One. 2018 Mar 29;13(3):e0194400. doi: 10.1371/journal.pone.0194400 (PMC5875757; doi:10.1371/journal.pone.0194400)
Supplement: S1 File — (DOCX) [file pone.0194400.s001.docx]

**Supporting information**

Analyses of genome wide association data, cytokines, and gene expression in neutrophils of African-Americans with benign ethnic neutropenia

Bashira A. Charles^1¶^, Matthew M. Hsieh^2¶^, Adebowale A. Adeyemo^1¶^, Daniel Shriner^1^, Edward Ramos^1,3^, Kyung Chin^2^, Kshitij Srivastava^4^, Neil A. Zakai^5^, Mary Cushman^5^, Leslie A. McClure^6,7^, Virginia Howard^6^, Willy A. Flegel^4^, Charles N. Rotimi^1^*, Griffin P. Rodgers^2^*

^1^Center for Research on Genomics and Global Health, National Human Genome Research Institute, National Institutes of Health (NIH), Bethesda, Maryland, USA; ^2^Molecular and Clinical Hematology Branch, National Institute of Diabetes and Digestive and Kidney Diseases, NIH, Bethesda, Maryland, USA; ^3^National Institute of Biomedical Imaging and Bioengineering, NIH, Bethesda, Maryland, USA; ^4^Warren Grant Magnuson Clinical Center, NIH, Bethesda, Maryland, USA; ^5^Departments of Pathology and Medicine, University of Vermont Larner College of Medicine, Burlington, Vermont, USA; ^6^ School of Public Health, University of Alabama, Birmingham, Alabama, USA; ^7^Department of Epidemiology and Biostatistics, Drexel University, Philadelphia, Pennsylvania, USA

^¶^These authors contributed equally to the work

*Corresponding Authors

E-mail: [rotimic@mail.nih.gov](mailto:rotimic@mail.nih.gov) (CNR) or [gr5n@ nih.gov](mailto:GriffinR@extra.niddk.nih.gov) (GPR)

Table 1. Characteristics of the discovery GWAS subjects (the REGARDS study)

|  | Low WBC (n=592) | | High WBC (n=586) | |
| --- | --- | --- | --- | --- |
|  | Median | IQR | Median | IQR |
| Age (years) | 62 | 14 | 62 | 13 |
| WBC (10^3^/uL) | 2.97 | 0.51 | 8.01 | 0.708 |
| Platelets (10^3^/uL) | 206 | 77 | 255 | 89 |
| C-Reactive Protein | 1.66 | 2.93 | 4.58 | 7.08 |
| Smoking (n, %) | 230 | 39.12 | 354 | 60.7 |

Table 2. Characteristics of the replication GWAS subjects (the ARIC study)

|  | Low WBC (n=191) |  | High WBC (n=628) |  |
| --- | --- | --- | --- | --- |
|  | Median | IQR | Median | IQR |
| Age (years) | 53 | 9 | 52 | 10 |
| WBC (10^3^/uL) | 3.2 | 0.5 | 7.5 | 1.5 |
| Smoking (n, %) | 101 | 52.88% | 290 | 46.18% |

Table 3. Characteristics of the subjects in the gene expression study

| Characteristic | BEN (ANC <1500/mm^3^)* | Non-BEN (ANC >4000/mm^3^) |
| --- | --- | --- |
| Sex (Male/Female) | 4/2 | 2/3 |
| Age in years [median(range)] | 44 (34-51) | 34 (24-38) |

*After exclusion of one outlier

Table 4. Top hits in the discovery GWAS (the REGARDS study)

| **SNP** | **CHR** | **BP** | **A1** | **FRQ** | **OR** | **SE** | **P** | **fxn_class** | **Gene symbol** |
| --- | --- | --- | --- | --- | --- | --- | --- | --- | --- |
| rs2814778 | 1 | 159174683 | T | 0.2084 | 0.0641 | 0.1791 | 4.09E-53 | upstream-2KB,  utr-5-prime | *DARC* |
| rs856046 | 1 | 158987941 | A | 0.8292 | 9.6175 | 0.1704 | 2.89E-40 | intron | *IFI16* |
| rs11577338 | 1 | 155240077 | G | 0.8502 | 5.5492 | 0.1615 | 2.66E-26 | intron | *CLK2* |
| rs6696888 | 1 | 155508882 | G | 0.8535 | 5.2763 | 0.1598 | 2.32E-25 | intron | *ASH1L* |
| rs4074436 | 1 | 154531910 | G | 0.7314 | 3.1202 | 0.1127 | 5.84E-24 | upstream-2KB | *UBE2Q1* |
| rs670523 | 1 | 155878732 | A | 0.825 | 4.3311 | 0.1456 | 7.45E-24 | intron | *RIT1* |
| rs863000 | 1 | 159170986 | T | 0.8754 | 6.5257 | 0.1887 | 2.77E-23 | nc-transcript,  utr-3-prime | *LOC100131825* |
| rs2501339 | 1 | 159825061 | G | 0.1326 | 0.1908 | 0.1706 | 2.74E-22 | intron,  utr-5-prime | *C1orf204* |
| rs10908720 | 1 | 159408803 | C | 0.8242 | 3.6981 | 0.1354 | 4.54E-22 | upstream-2KB | *OR10J1* |
| rs6699071 | 1 | 157494812 | A | 0.6166 | 2.4597 | 0.098 | 4.33E-20 | intron | *FCRL5* |
| rs12723848 | 1 | 162237979 | G | 0.8686 | 4.2674 | 0.1583 | 4.79E-20 | intron | *NOS1AP* |
| rs4845700 | 1 | 154981708 | C | 0.681 | 2.5427 | 0.1019 | 5.33E-20 | intron | *ZBTB7B* |
| rs7516146 | 1 | 159801111 | G | 0.8937 | 5.4433 | 0.1866 | 1.10E-19 | intron | *SLAMF8* |
| rs855871 | 1 | 159044651 | G | 0.6118 | 2.4589 | 0.0992 | 1.19E-19 | intron | *AIM2* |
| rs12025136 | 1 | 161980502 | C | 0.7874 | 2.9459 | 0.1196 | 1.69E-19 | intron | *OLFML2B* |
| rs6682716 | 1 | 156551848 | A | 0.8104 | 3.2059 | 0.1291 | 1.79E-19 | missense, reference | *TTC24* |
| rs411088 | 1 | 158171295 | C | 0.6337 | 2.4241 | 0.0989 | 3.55E-19 | intron | *LOC100505799* |
| rs2789424 | 1 | 159942456 | C | 0.728 | 2.6154 | 0.1079 | 5.26E-19 | intron | *LOC100505633* |
| rs1065457 | 1 | 158324425 | A | 0.1883 | 0.3261 | 0.1277 | 1.71E-18 | intron, missense | *CD1E* |
| rs11576266 | 1 | 156572159 | A | 0.8024 | 2.9652 | 0.1253 | 4.07E-18 | upstream-2KB | *GPATCH4* |
| rs4845490 | 1 | 152849299 | A | 0.3591 | 0.4231 | 0.0993 | 4.80E-18 | upstream-2KB | *SMCP* |
| rs1704745 | 1 | 162693795 | A | 0.8538 | 3.6471 | 0.1502 | 7.15E-18 | intron | *DDR2* |
| rs1633276 | 1 | 158903815 | C | 0.4181 | 2.9144 | 0.1248 | 1.02E-17 | intron | *PYHIN1* |
| rs942679 | 1 | 156351066 | C | 0.8852 | 4.322 | 0.1718 | 1.57E-17 | intron | *RHBG* |
| rs7529060 | 1 | 157725264 | T | 0.1401 | 0.2777 | 0.1504 | 1.61E-17 | intron | *FCRL2* |
| rs539118 | 1 | 150966197 | G | 0.708 | 2.5263 | 0.1089 | 1.69E-17 | intron | *ANXA9* |
| rs2249707 | 1 | 159922959 | C | 0.6887 | 2.4582 | 0.1057 | 1.71E-17 | intron | *SLAMF9* |
| rs12401997 | 1 | 156147796 | C | 0.9099 | 5.8241 | 0.208 | 2.45E-17 | downstream-500B | *SEMA4A* |
| rs1320489 | 1 | 157977491 | C | 0.925 | 9.2075 | 0.2627 | 2.92E-17 | intron | *KIRREL* |
| rs505058 | 1 | 156106185 | T | 0.3937 | 0.45 | 0.0945 | 3.00E-17 | synonymous- codon | *LMNA* |

Table 5. Top hits in the discovery GWAS after conditioning on leading *DARC* SNP (the REGARDS study)

|  | **SNP** | **Chr** | **A1** | **A2** | **FRQ** | **OR** | **SE** | **P** | **Class** | **Gene symbol** |
| --- | --- | --- | --- | --- | --- | --- | --- | --- | --- | --- |
| 1 | rs77998448 | 7 | A | G | 0.928 | 2.8926 | 0.2017 | 1.40E-07 | intergenic |  |
| 2 | rs77255239 | 7 | A | G | 0.9277 | 2.8892 | 0.2017 | 1.45E-07 | intergenic |  |
| 3 | rs73068906 | 5 | C | A | 0.9375 | 3.3183 | 0.2313 | 2.16E-07 | intron | *CDH12 (CDHB)* |
| 4 | rs866991 | 9 | G | A | 0.3391 | 0.5612 | 0.1135 | 3.62E-07 | intergenic |  |
| 5 | rs77696237 | 5 | A | C | 0.9372 | 3.2145 | 0.2296 | 3.65E-07 | intron | *CDH12* |
| 6 | rs4741700 | 9 | A | G | 0.3394 | 0.5612 | 0.1137 | 3.71E-07 | intergenic |  |
| 7 | rs3950913 | 5 | A | G | 0.9387 | 3.452 | 0.2453 | 4.38E-07 | intron | *CDH12* |
| 8 | rs115261301 | 5 | T | A | 0.9065 | 2.6891 | 0.1986 | 6.33E-07 | intron | *CDH12* |
| 9 | rs2552473 | 18 | C | A | 0.4285 | 1.7161 | 0.1104 | 9.95E-07 | intergenic |  |
| 10 | rs114883666 | 5 | G | A | 0.9261 | 2.8026 | 0.2118 | 1.14E-06 | intron | *CDH12* |
| 11 | rs76939696 | 7 | T | G | 0.9471 | 3.5701 | 0.2618 | 1.17E-06 | intergenic |  |
| 12 | rs114086506 | 1 | G | A | 0.9713 | 0.1391 | 0.4084 | 1.36E-06 | intergenic |  |
| 13 | rs56761758 | 5 | A | G | 0.9448 | 3.3942 | 0.2532 | 1.39E-06 | intron | *CDH12* |
| 14 | rs10966132 | 9 | G | C | 0.3762 | 0.5931 | 0.1087 | 1.53E-06 | intergenic |  |
| 15 | rs113407698 | 7 | G | A | 0.9227 | 2.5454 | 0.1954 | 1.75E-06 |  |  |
| 16 | rs111736039 | 7 | G | A | 0.9223 | 2.5348 | 0.1952 | 1.90E-06 | intergenic |  |
| 17 | rs2358581 | 19 | T | G | 0.449 | 0.5776 | 0.1153 | 1.95E-06 | intron | *ICAM1 (BB2 CD54 P3.58)* |
| 18 | rs78717017 | 7 | G | A | 0.9218 | 2.5216 | 0.1949 | 2.07E-06 | intergenic |  |
| 19 | rs17081115 | 4 | G | A | 0.9411 | 3.02 | 0.2342 | 2.36E-06 | intergenic |  |
| 20 | rs75178228 | 7 | T | C | 0.9205 | 2.4805 | 0.1925 | 2.37E-06 | intergenic |  |

Table 6. Duffy phenotype frequencies computed from genotype allele frequencies in the REGARDS study

| Phenotype | c.125G>A  rs12075 | c.1-67T>C  rs2814778 | Frequencies (%) | | | |
| --- | --- | --- | --- | --- | --- | --- |
|  |  |  | Low WBC | High WBC | Africans* | European* |
| Fy(a+b-) | G/G  G/A | T/T  T/C | 3.5 | 24.6 | 10 | 20 |
| Fy(a+b+) | G/A | T/T | 0.5 | 2.6 | 3 | 48 |
| Fy(a-b+) | A/A  A/A | T/T  T/C | 5.0 | 36.0 | 20 | 32 |
| Fy(a-b-) | A/A | C/C | 91.0 | 36.8 | 67 | Rare |

*After Mourant et al (monograph: Distribution of Human Blood Groups, 1976) and De Silva et al (PLoS ONE 2014)

Table 7. Top hits in replication GWAS (the ARIC study)

| **Chr** | **SNP** | **CHR** | **BP** | **A1** | **A2** | **FRQ** | **OR** | **SE** | **P** | **GENE** |
| --- | --- | --- | --- | --- | --- | --- | --- | --- | --- | --- |
| 1 | rs2814778 | 1 | 159174683 | T | C | 0.2304 | 0.0226 | 0.4052 | 8.24E-21 | *DARC* |
| 2 | rs2570916 | 1 | 159012646 | C | T | 0.7872 | 10.7401 | 0.2726 | 3.11E-18 | *IFI16* |
| 3 | rs2852699 | 1 | 159018359 | G | C | 0.7751 | 9.6452 | 0.261 | 3.83E-18 | *IFI16* |
| 4 | rs855866 | 1 | 159052720 | A | G | 0.8062 | 21.472 | 0.3565 | 7.78E-18 | *AIM2* |
| 5 | rs1894043 | 1 | 159069211 | T | C | 0.8009 | 45.4118 | 0.4444 | 9.04E-18 | *AIM2* |
| 6 | rs856068 | 1 | 158983777 | T | C | 0.7908 | 12.1219 | 0.2951 | 2.77E-17 | *IFI16* |
| 7 | rs856066 | 1 | 158983593 | C | G | 0.787 | 10.0855 | 0.2787 | 1.12E-16 | *IFI16* |
| 8 | rs2084257 | 1 | 159523486 | C | A | 0.7289 | 5.9238 | 0.2197 | 5.65E-16 | *OR10J5* |
| 9 | rs2518564 | 1 | 159062436 | G | A | 0.8265 | 38.0887 | 0.4502 | 6.22E-16 | *AIM2* |
| 10 | rs2518563 | 1 | 159059969 | C | T | 0.8268 | 39.4879 | 0.4551 | 6.66E-16 | *AIM2* |
| 11 | rs2789423 | 1 | 159885500 | G | A | 0.8061 | 13.7965 | 0.3388 | 9.54E-15 | *TAGLN2* |
| 12 | rs2501314 | 1 | 159882077 | G | A | 0.7182 | 6.0498 | 0.234 | 1.46E-14 | *TAGLN2* |
| 13 | rs856046 | 1 | 158987941 | A | G | 0.82 | 14.5492 | 0.3505 | 2.19E-14 | *IFI16* |
| 14 | rs4339862 | 1 | 159441628 | C | A | 0.739 | 5.1469 | 0.2159 | 3.20E-14 | *OR10J1* |
| 15 | rs1633276 | 1 | 158903815 | C | T | 0.3453 | 6.4494 | 0.2463 | 3.82E-14 | *PYHIN1* |
| 16 | rs35615695 | 1 | 155501014 | T | C | 0.8554 | 23.6099 | 0.4215 | 6.32E-14 | *ASH1L* |
| 17 | rs11265157 | 1 | 159224029 | C | G | 0.2163 | 0.1642 | 0.2411 | 6.65E-14 | *FCER1A* |
| 18 | rs7537391 | 1 | 159223587 | G | A | 0.202 | 0.1473 | 0.2557 | 6.76E-14 | *FCER1A* |
| 19 | rs6692378 | 1 | 159339241 | T | C | 0.1842 | 0.14 | 0.2629 | 7.59E-14 | *OR10J3* |
| 20 | rs12145079 | 1 | 159406538 | G | A | 0.8178 | 7.2401 | 0.265 | 7.93E-14 | *OR10J1* |
| 21 | rs7542509 | 1 | 159349465 | A | C | 0.1843 | 0.1409 | 0.2623 | 7.94E-14 | *OR10J1* |
| 22 | rs3122631 | 1 | 159362517 | T | C | 0.1843 | 0.1414 | 0.2621 | 8.44E-14 | *OR10J1* |
| 23 | rs856043 | 1 | 158987241 | A | G | 0.7932 | 9.7125 | 0.3047 | 8.50E-14 | *IFI16* |
| 24 | rs3122629 | 1 | 159363819 | T | C | 0.1843 | 0.1416 | 0.262 | 8.54E-14 | *OR10J1* |
| 25 | rs3131547 | 1 | 159364301 | A | G | 0.1843 | 0.1416 | 0.262 | 8.61E-14 | *OR10J1* |
| 26 | rs3122628 | 1 | 159366274 | C | T | 0.1843 | 0.1417 | 0.2621 | 8.91E-14 | *OR10J1* |
| 27 | rs1320568 | 1 | 159912346 | A | G | 0.8295 | 19.6134 | 0.4004 | 1.06E-13 | *IGSF9* |
| 28 | rs12034969 | 1 | 159384944 | T | C | 0.8176 | 7.0043 | 0.263 | 1.34E-13 | *OR10J1* |
| 29 | rs2768762 | 1 | 156860462 | G | T | 0.7973 | 6.8863 | 0.2612 | 1.51E-13 | *PEAR1* |
| 30 | rs7546146 | 1 | 159225892 | G | C | 0.2465 | 0.1867 | 0.2274 | 1.58E-13 | *FCER1A* |
| 31 | rs55872368 | 1 | 159176856 | G | T | 0.2765 | 0.1924 | 0.2235 | 1.65E-13 | *DARC* |
| 32 | rs34632540 | 1 | 155612197 | G | T | 0.8593 | 21.8141 | 0.418 | 1.65E-13 | *MSTO1* |
| 33 | rs4971072 | 1 | 155273869 | G | A | 0.8562 | 23.8064 | 0.4306 | 1.81E-13 | *HCN3* |
| 34 | rs1101988 | 1 | 158992195 | A | G | 0.7919 | 8.8256 | 0.2961 | 1.92E-13 | *IFI16* |
| 35 | rs4517343 | 1 | 159339006 | A | G | 0.2036 | 0.1686 | 0.2421 | 1.93E-13 | *OR10J3* |
| 36 | rs4537549 | 1 | 159339036 | C | G | 0.2036 | 0.1687 | 0.2421 | 1.94E-13 | *OR10J3* |
| 37 | rs6692609 | 1 | 159339463 | T | C | 0.2036 | 0.1687 | 0.242 | 1.94E-13 | *OR10J3* |
| 38 | rs6677678 | 1 | 159339520 | C | T | 0.2036 | 0.1687 | 0.242 | 1.94E-13 | *OR10J3* |
| 39 | rs10908713 | 1 | 159339559 | T | C | 0.2036 | 0.1687 | 0.242 | 1.94E-13 | *OR10J3* |
| 40 | rs4400605 | 1 | 159340416 | T | C | 0.2036 | 0.1688 | 0.242 | 1.96E-13 | *OR10J3* |
| 41 | rs11582072 | 1 | 155477570 | T | C | 0.8586 | 22.9191 | 0.4263 | 2.04E-13 | *ASH1L* |
| 42 | rs10908720 | 1 | 159408803 | C | T | 0.8158 | 6.6211 | 0.2574 | 2.07E-13 | *OR10J1* |
| 43 | rs6696888 | 1 | 155508882 | G | A | 0.8589 | 23.0104 | 0.427 | 2.08E-13 | *ASH1L* |
| 44 | rs1101997 | 1 | 158998254 | C | A | 0.8022 | 9.1549 | 0.3017 | 2.14E-13 | *IFI16* |
| 45 | rs4656926 | 1 | 160650913 | A | G | 0.4388 | 5.5483 | 0.234 | 2.41E-13 | *CD48* |
| 46 | rs7548736 | 1 | 159340686 | A | G | 0.2027 | 0.1717 | 0.2411 | 2.72E-13 | *OR10J3* |
| 47 | rs6687840 | 1 | 159342439 | C | T | 0.2027 | 0.1719 | 0.241 | 2.74E-13 | *OR10J3* |
| 48 | rs4492611 | 1 | 156663217 | A | G | 0.7124 | 9.0466 | 0.3016 | 2.83E-13 | *CRABP2* |
| 49 | rs11265158 | 1 | 159224030 | T | C | 0.2105 | 0.1758 | 0.2384 | 3.04E-13 | *FCER1A* |
| 50 | rs6665483 | 1 | 159342733 | G | A | 0.2062 | 0.1644 | 0.2477 | 3.09E-13 | *OR10J3* |
| 51 | rs857859 | 1 | 158771450 | T | A | 0.1621 | 0.0797 | 0.3471 | 3.16E-13 | *OR6N2* |
| 52 | rs436361 | 1 | 158205376 | T | C | 0.6629 | 4.0654 | 0.1929 | 3.55E-13 | *CD1A* |
| 53 | rs11812028 | 1 | 161974641 | A | T | 0.8512 | 26.8562 | 0.4531 | 3.80E-13 | *OLFML2B* |
| 54 | rs4446959 | 1 | 159357684 | T | C | 0.2039 | 0.1794 | 0.237 | 4.13E-13 | *OR10J1* |
| 55 | rs1934073 | 1 | 159936733 | T | G | 0.8613 | 32.7531 | 0.4812 | 4.16E-13 | *LINC01133* |
| 56 | rs822478 | 1 | 155793969 | T | C | 0.8541 | 14.5818 | 0.3702 | 4.50E-13 | *GON4L* |
| 57 | rs59012264 | 1 | 159251511 | A | G | 0.2145 | 0.1914 | 0.2294 | 5.73E-13 | *FCER1A* |
| 58 | rs60932309 | 1 | 159228924 | T | C | 0.2135 | 0.1823 | 0.2362 | 5.76E-13 | *FCER1A* |
| 59 | rs2325920 | 1 | 159226421 | T | C | 0.2089 | 0.1831 | 0.2359 | 6.19E-13 | *FCER1A* |
| 60 | rs10908702 | 1 | 159228378 | C | T | 0.2088 | 0.1832 | 0.2359 | 6.29E-13 | *FCER1A* |
| 61 | rs3122630 | 1 | 159363731 | T | C | 0.1896 | 0.167 | 0.2488 | 6.31E-13 | *OR10J1* |
| 62 | rs2789427 | 1 | 159953315 | T | C | 0.6384 | 4.7531 | 0.2169 | 6.72E-13 | *LINC01133* |
| 63 | rs429201 | 1 | 158194899 | C | T | 0.7197 | 4.1703 | 0.199 | 7.12E-13 | *CD1A* |
| 64 | rs6427381 | 1 | 157453883 | G | A | 0.7285 | 5.0684 | 0.2267 | 8.02E-13 | *FCRL5* |
| 65 | rs821551 | 1 | 155688580 | C | A | 0.1334 | 0.0484 | 0.4237 | 8.73E-13 | *DAP3* |
| 66 | rs4656347 | 1 | 161996062 | T | C | 0.8489 | 10.2889 | 0.3262 | 8.92E-13 | *OLFML2B* |
| 67 | rs2325924 | 1 | 159233756 | T | C | 0.2114 | 0.1928 | 0.2306 | 9.53E-13 | *FCER1A* |
| 68 | rs10918438 | 1 | 161976817 | T | G | 0.8283 | 7.9938 | 0.2913 | 9.69E-13 | *OLFML2B* |
| 69 | rs11264422 | 1 | 155907823 | T | A | 0.8506 | 10.2715 | 0.3265 | 9.76E-13 | *KIAA0907* |
| 70 | rs10908714 | 1 | 159340353 | T | A | 0.8576 | 12.1515 | 0.3512 | 1.14E-12 | *OR10J3* |
| 71 | rs11265190 | 1 | 159391401 | G | A | 0.8096 | 5.6758 | 0.2449 | 1.36E-12 | *OR10J1,* |
| 72 | rs11582600 | 1 | 159357373 | C | T | 0.8579 | 12.0026 | 0.3507 | 1.39E-12 | *OR10J1* |
| 73 | rs4657136 | 1 | 161994010 | G | T | 0.8492 | 9.3653 | 0.3165 | 1.57E-12 | *OLFML2B* |
| 74 | rs6664820 | 1 | 158906416 | G | T | 0.8335 | 12.6078 | 0.3588 | 1.62E-12 | *PYHIN1* |
| 75 | rs2852727 | 1 | 159086986 | G | A | 0.7953 | 6.7343 | 0.2702 | 1.67E-12 | *AIM2* |
| 76 | rs1894044 | 1 | 159088334 | G | C | 0.7954 | 6.7284 | 0.27 | 1.67E-12 | *AIM2* |
| 77 | rs670523 | 1 | 155878732 | A | G | 0.8537 | 10.3381 | 0.3309 | 1.68E-12 | *RIT1* |
| 78 | rs1122609 | 1 | 159089715 | T | C | 0.7956 | 6.7312 | 0.2701 | 1.68E-12 | *AIM2* |
| 79 | rs6676438 | 1 | 161983089 | T | C | 0.8471 | 9.2666 | 0.3161 | 1.86E-12 | *OLFML2B* |
| 80 | rs3811035 | 1 | 157485561 | G | A | 0.7845 | 5.9437 | 0.2532 | 1.93E-12 | *FCRL5* |
| 81 | rs9427342 | 1 | 159360156 | C | G | 0.2851 | 0.231 | 0.2086 | 2.16E-12 | *OR10J1* |
| 82 | rs6675789 | 1 | 159430112 | A | T | 0.7234 | 4.0708 | 0.2001 | 2.29E-12 | *OR10J1* |
| 83 | rs12566012 | 1 | 159426952 | C | T | 0.712 | 4.1105 | 0.2017 | 2.44E-12 | *OR10J1* |
| 84 | rs1103577 | 1 | 159100315 | T | C | 0.8108 | 7.1639 | 0.2815 | 2.66E-12 | *AIM2* |
| 85 | rs2073657 | 1 | 161010791 | C | T | 0.8209 | 5.9494 | 0.255 | 2.69E-12 | *USF1* |
| 86 | rs4454545 | 1 | 159304157 | G | A | 0.345 | 0.2783 | 0.183 | 2.76E-12 | *OR10J3* |
| 87 | rs856049 | 1 | 158990412 | A | G | 0.7214 | 5.432 | 0.2422 | 2.82E-12 | *IFI16* |
| 88 | rs4656344 | 1 | 161990455 | T | G | 0.841 | 7.7628 | 0.2934 | 2.84E-12 | *OLFML2B* |
| 89 | rs2789422 | 1 | 159892088 | G | A | 0.8577 | 19.7896 | 0.4277 | 2.97E-12 | *TAGLN2* |
| 90 | rs2820187 | 1 | 158903252 | G | A | 0.1754 | 0.07 | 0.3816 | 3.22E-12 | *PYHIN1* |
| 91 | rs490498 | 1 | 155880952 | G | A | 0.8337 | 6.9979 | 0.2798 | 3.56E-12 | *RIT1* |
| 92 | rs2768759 | 1 | 156852463 | A | C | 0.8128 | 6.9264 | 0.2783 | 3.57E-12 | *NTRK1* |
| 93 | rs2188118 | 1 | 159075696 | G | C | 0.7704 | 6.2322 | 0.2634 | 3.75E-12 | *AIM2* |
| 94 | rs863326 | 1 | 158640513 | T | C | 0.5505 | 3.1514 | 0.1659 | 4.53E-12 | *SPTA1* |
| 95 | rs1102009 | 1 | 158925719 | C | T | 0.6726 | 4.1634 | 0.2065 | 4.96E-12 | *PYHIN1* |
| 96 | rs7523246 | 1 | 161972767 | G | A | 0.8343 | 11.1481 | 0.3495 | 5.21E-12 | *OLFML2B* |
| 97 | rs7535596 | 1 | 157673356 | A | G | 0.2425 | 0.2421 | 0.2057 | 5.42E-12 | *FCRL3* |
| 98 | rs34388918 | 1 | 159416725 | G | A | 0.8558 | 9.8088 | 0.3316 | 5.75E-12 | *OR10J1* |
| 99 | rs12023055 | 1 | 159418008 | A | G | 0.8557 | 9.718 | 0.3306 | 6.05E-12 | *OR10J1* |
| 100 | rs12133577 | 1 | 159432278 | C | T | 0.8555 | 9.6697 | 0.3301 | 6.26E-12 | *OR10J1* |

Table 8. Association p-values for variants in the *EPHA3* gene in the replication (ARIC) study

| **rsID** | **REGARDS OR** | **REGARDS p-value** | **ARIC OR** | **ARIC p-value** |
| --- | --- | --- | --- | --- |
| rs36076607 | 1.6133 | 4.67E-08 | 1.0228 | 0.8708 |
| rs11929346 | 1.6008 | 7.26E-08 | 1.0354 | 0.8007 |
| rs35800684 | 1.6004 | 7.28E-08 | 1.0363 | 0.796 |
| rs34046877 | 1.638 | 7.79E-08 | 0.9313 | 0.6264 |
| rs6794198 | 1.6378 | 7.86E-08 | 0.9313 | 0.6262 |
| rs34519192 | 1.6274 | 7.88E-08 | 0.9192 | 0.5587 |
| rs11920519 | 1.6273 | 7.90E-08 | 0.9191 | 0.5583 |
| rs1996069 | 1.6328 | 8.24E-08 | 0.9467 | 0.7055 |
| rs36076607 | 1.6133 | 4.67E-08 | 1.0228 | 0.8708 |
| rs34519192 | 1.6274 | 7.88E-08 | 0.9192 | 0.5587 |
| rs17026797 | 1.6471 | 8.63E-08 | 0.9334 | 0.6376 |
| rs7629132 | 1.6237 | 9.06E-08 | 0.9147 | 0.5361 |
| rs73015088* | 0.9712 | 0.7755 | 2.2836 | 1.26E-05 |
| rs479285* | 0.8353 | 0.1649 | 0.3269 | 1.29E-05 |

*Lowest p-value in *EPHA3* in ARIC

Table 9. Gene level (full-length transcript) differential expression between BEN and non-BEN individuals

| **Transcript ID** | **Gene Symbol** | **RefSeq** | **p-value (BEN vs. Control)** | **Ratio (BEN vs. Control)** | **Fold-change (BEN vs. Control)** | **Fold-change (BEN vs. Control) (Description)** |
| --- | --- | --- | --- | --- | --- | --- |
| 16863691 | *CRX* | ENST00000221996 | 1.04E-06 | 0.74006 | -1.351 | BEN down vs Control |
| 16778849 | *LCP1* | NM_002298 | 6.54E-05 | 0.78486 | -1.274 | BEN down vs Control |
| 16837065 | *CEP95* | ENST00000556440 | 8.30E-05 | 1.6499 | 1.649 | BEN up vs Control |
| 16770344 | *HECTD4* | NM_001109662 | 9.37E-05 | 1.14173 | 1.141 | BEN up vs Control |
| 17068938 | *RGS20* | NM_170587 | 9.59E-05 | 0.82088 | -1.218 | BEN down vs Control |
| 16914395 | *MMP9* | NM_004994 | 0.00013 | 0.55232 | -1.810 | BEN down vs Control |
| 16898788 | *TGFA* | NM_003236 | 0.00020 | 0.71191 | -1.404 | BEN down vs Control |
| 16855684 | *KDSR* | ENST00000406396 | 0.00022 | 1.59934 | 1.599 | BEN up vs Control |
| 16860302 | *ZNF91* | ENST00000595893 | 0.00026 | 1.383 | 1.383 | BEN up vs Control |
| 16771146 | *GCN1L1* | ENST00000300648 | 0.00027 | 1.38113 | 1.381 | BEN up vs Control |
| 16738174 | *FNBP4* | NM_015308 | 0.00028 | 1.45675 | 1.456 | BEN up vs Control |
| 16664708 | *ZFYVE9* | NM_004799 | 0.00029 | 1.5275 | 1.527 | BEN up vs Control |
| 16928938 | *SEC14L2* | NM_033382 | 0.00031 | 1.47769 | 1.477 | BEN up vs Control |
| 16722324 | *CALCB* | NM_000728 | 0.00037 | 1.3669 | 1.366 | BEN up vs Control |
| 16797751 | *TUBGCP5* | NM_052903 | 0.00038 | 1.37467 | 1.374 | BEN up vs Control |
| 17028297 | *RNF5* | AK296212 | 0.00039 | 1.17123 | 1.171 | BEN up vs Control |
| 16982936 | *LOC255167* | NR_024423 | 0.00040 | 0.77618 | -1.288 | BEN down vs Control |
| 16876103 | *ZNF329* | NM_024620 | 0.00040 | 1.18324 | 1.183 | BEN up vs Control |
| 16977364 | *HNRNPDL* | NR_003249 | 0.00043 | 1.32233 | 1.322 | BEN up vs Control |
| 17030833 | *HSPA1B* | NM_005346 | 0.00046 | 0.79344 | -1.260 | BEN down vs Control |
| 17028007 | *HSPA1B* | NM_005346 | 0.00053 | 0.75724 | -1.320 | BEN down vs Control |

Table 10. Hematologic/Immune pathways enriched in top genes showing differential full-length transcript expression

| **#** | **Hem/Immune System Diseases Maps** | **Total objects** | **p-value** | **FDR** | **Network Objects from Active Data** |
| --- | --- | --- | --- | --- | --- |
| 1 | Development_Role of proteases in hematopoietic stem cell mobilization | 18 | 2.354E-02 | 7.863E-02 | MMP-9 |
| 2 | Development_Role of G-CSF in hematopoietic stem cell mobilization | 21 | 2.743E-02 | 7.863E-02 | MMP-9 |
| 3 | Development_Role of HGF in hematopoietic stem cell mobilization | 21 | 2.743E-02 | 7.863E-02 | MMP-9 |
| 4 | Proteolysis_Putative ubiquitin pathway | 23 | 3.002E-02 | 7.863E-02 | HSP70 |
| 5 | Chemotaxis_CCL19- and CCl21-mediated chemotaxis | 33 | 4.294E-02 | 7.863E-02 | MMP-9 |
| 6 | Immune response_TLR ligands | 34 | 4.422E-02 | 7.863E-02 | HSP70 |
| 7 | Development_SDF-1 signaling in hematopoietic stem cell homing | 38 | 4.936E-02 | 7.863E-02 | MMP-9 |
| 8 | "Chemotaxis_CCL16- CCL20- CXCL16- and CCL25-mediated cell migration" | 43 | 5.576E-02 | 7.863E-02 | MMP-9 |
| 9 | Immune response_Histamine H1 receptor signaling in immune response | 48 | 6.214E-02 | 7.863E-02 | MMP-9 |
| 10 | Immune response_HSP60 and HSP70/ TLR signaling pathway | 54 | 6.977E-02 | 7.863E-02 | HSP70 |

Table 11. Pathways enriched in top genes showing differential full-length transcript expression

| **#** | **Maps** | **Total objects** | **p-Value** | **FDR** | **Network Objects from Active Data** |
| --- | --- | --- | --- | --- | --- |
| 1 | Regulation of degradation of deltaF508-CFTR in CF | 39 | 3.632E-04 | 2.107E-02 | HSP70, RNF5 |
| 2 | Development_EGFR signaling pathway | 71 | 1.204E-03 | 3.492E-02 | MMP-9, TGF-alpha |
| 3 | Development_Role of proteases in hematopoietic stem cell mobilization | 18 | 1.363E-02 | 5.127E-02 | MMP-9 |
| 4 | Regulation of degradation of wtCFTR | 20 | 1.513E-02 | 5.127E-02 | RNF5 |
| 5 | Development_Role of G-CSF in hematopoietic stem cell mobilization | 21 | 1.588E-02 | 5.127E-02 | MMP-9 |
| 6 | Development_Role of HGF in hematopoietic stem cell mobilization | 21 | 1.588E-02 | 5.127E-02 | MMP-9 |
| 7 | Proteolysis_Putative ubiquitin pathway | 23 | 1.738E-02 | 5.127E-02 | HSP70 |
| 8 | CFTR folding and maturation (normal and CF) | 24 | 1.813E-02 | 5.127E-02 | HSP70 |
| 9 | Development_Glucocorticoid receptor signaling | 24 | 1.813E-02 | 5.127E-02 | HSP70 |
| 10 | Proteolysis_Role of Parkin in the Ubiquitin-Proteasomal Pathway | 24 | 1.813E-02 | 5.127E-02 | HSP70 |
| 11 | Huntingtin-depended transcription deregulation in Huntington's Disease | 24 | 1.813E-02 | 5.127E-02 | HSPA1A |
| 12 | Resolution of inflammation in healing myocardial infarction | 25 | 1.888E-02 | 5.127E-02 | MMP-9 |
| 13 | HCV-dependent regulation of membrane receptors signaling in HCC | 27 | 2.038E-02 | 5.127E-02 | TGF-alpha |
| 14 | Transcription_Role of Akt in hypoxia induced HIF1 activation | 27 | 2.038E-02 | 5.127E-02 | HSP70 |
| 15 | Apoptosis and survival_Role of IAP-proteins in apoptosis | 31 | 2.337E-02 | 5.127E-02 | HSP70 |
| 16 | Chemotaxis_CCL19- and CCl21-mediated chemotaxis | 33 | 2.486E-02 | 5.127E-02 | MMP-9 |
| 17 | Normal and pathological TGF-beta-mediated regulation of cell proliferation | 33 | 2.486E-02 | 5.127E-02 | SARA |
| 18 | Oxidative stress_Role of ASK1 under oxidative stress | 34 | 2.561E-02 | 5.127E-02 | HSP70 |
| 19 | Immune response_TLR ligands | 34 | 2.561E-02 | 5.127E-02 | HSP70 |
| 20 | Role of growth factor receptors transactivation by Hyaluronic acid / CD44 signaling in tumor progression | 35 | 2.635E-02 | 5.127E-02 | MMP-9 |
| 21 | HBV signaling via protein kinases leading to HCC | 36 | 2.710E-02 | 5.127E-02 | MMP-9 |
| 22 | Cell adhesion_Cell-matrix glycoconjugates | 38 | 2.858E-02 | 5.127E-02 | MMP-9 |
| 23 | Cell cycle_Regulation of G1/S transition (part 1) | 38 | 2.858E-02 | 5.127E-02 | SARA |
| 24 | Signal transduction_Soluble CXCL16 signaling | 38 | 2.858E-02 | 5.127E-02 | MMP-9 |
| 25 | Development_SDF-1 signaling in hematopoietic stem cell homing | 38 | 2.858E-02 | 5.127E-02 | MMP-9 |
| 26 | Development_ERBB-family signaling | 39 | 2.932E-02 | 5.127E-02 | TGF-alpha |
| 27 | Immune response_HMGB1 release from the cell | 41 | 3.081E-02 | 5.127E-02 | HSPA1A |

Table 12. Exon-level differential expression between BEN and non-BEN individuals

| **Probeset ID** | **exon_id** | **Gene Symbol** | **RefSeq** | **p-value (BEN vs. non-BEN)** | **Ratio (BEN vs. non-BEN)** | **Fold-change (BEN vs. non-BEN)** |
| --- | --- | --- | --- | --- | --- | --- |
| 16770143 | 5062052 | *PPP1CC* | NM_002710 | 2.12E-06 | 0.473298 | -2.11283 |
| 16807484 | 5090803 | *RMDN3* | NM_018145 | 8.11E-06 | 0.370739 | -2.69732 |
| 16697687 | 5007936 | *ZNF281* | NM_012482 | 1.20E-05 | 0.682709 | -1.46475 |
| 17069142 | 5294240 | *LOC286177* | NR_038874 | 1.49E-05 | 0.712997 | -1.40253 |
| 17080260 | 5302876 | *CSMD3* | NM_198123 | 1.72E-05 | 7.77386 | 7.77386 |
| 16652841 | 0 |  | --- | 2.01E-05 | 2.00492 | 2.00492 |
| 16732864 | 5035010 | *TBRG1* | ENST00000473629 | 2.29E-05 | 2.91525 | 2.91525 |
| 16816397 | 5097900 | *TMC7* | NM_024847 | 2.65E-05 | 2.49442 | 2.49442 |
| 17058624 | 5286118 | *RFC2* | ENST00000485545 | 2.69E-05 | 1.94017 | 1.94017 |
| 16821143 | 5101857 | *MON1B* | NM_014940 | 4.04E-05 | 2.26984 | 2.26984 |
| 16837446 | 5115040 | *COG1* | NM_018714 | 4.07E-05 | 1.30071 | 1.30071 |
| 16822989 | 5103315 |  | --- | 4.29E-05 | 2.38209 | 2.38209 |
| 16875065 | 5145718 | *ZNF83* | AB209516 | 4.66E-05 | 6.01721 | 6.01721 |
| 16857746 | 5131864 | *TRAPPC5* | NM_174894 | 4.74E-05 | 0.671081 | -1.49013 |
| 16935596 | 5192771 | *TCF20* | NM_005650 | 4.90E-05 | 1.50092 | 1.50092 |
| 16697190 | 5007525 | *EDEM3* | NM_025191 | 5.13E-05 | 0.473896 | -2.11017 |
| 16854567 | 5129169 | *TRAPPC8* | NM_014939 | 5.27E-05 | 0.593659 | -1.68447 |
| 16698535 | 5008582 | *NUCKS1* | NM_022731 | 5.36E-05 | 1.53308 | 1.53308 |
| 16770650 | 5062390 | *RBM19* | NM_001146699 | 5.52E-05 | 2.68383 | 2.68383 |
| 16958475 | 5209927 | *ZNF148* | NM_021964 | 5.56E-05 | 0.659809 | -1.51559 |
| 16724540 | 5029284 | *MADD* | NM_130470 | 5.61E-05 | 2.95983 | 2.95983 |
| 16869275 | 5141089 | *TNPO2* | NM_001136196 | 6.04E-05 | 1.67961 | 1.67961 |
| 17017178 | 5256015 | *ATP6V1G2-DDX39B* | NR_037853 | 6.20E-05 | 1.74963 | 1.74963 |
| 17029040 | 5264815 |  | --- | 6.20E-05 | 1.74963 | 1.74963 |
| 17041821 | 5273215 |  | --- | 6.20E-05 | 1.74963 | 1.74963 |
| 17050141 | 5279563 | *PIK3CG* | NM_002649 | 6.55E-05 | 0.68238 | -1.46546 |
| 17069514 | 5294546 | *DNAJC5B* | NM_033105 | 6.84E-05 | 0.421386 | -2.37312 |
| 16729581 | 5032595 |  | --- | 6.96E-05 | 0.478737 | -2.08883 |
| 16774553 | 5065435 | *COG3* | NM_031431 | 7.48E-05 | 1.54729 | 1.54729 |
| 16953162 | 5206035 | *SCAP* | ENST00000416847 | 7.56E-05 | 7.42627 | 7.42627 |
| 17122009 | 0 |  | --- | 7.82E-05 | 1.43272 | 1.43272 |
| 16880926 | 5150394 | *PNO1* | NM_020143 | 8.02E-05 | 2.22981 | 2.22981 |
| 17065916 | 5291790 | *KIAA1456* | ENST00000528335 | 8.23E-05 | 1.73423 | 1.73423 |
| 17117117 | 5331648 | *BCORP1* | NR_033732 | 8.29E-05 | 2.62707 | 2.62707 |
| 16960630 | 5211570 |  | --- | 8.31E-05 | 0.546979 | -1.82823 |
| 16705548 | 5014296 | *SUPV3L1* | NM_003171 | 8.44E-05 | 2.64502 | 2.64502 |
| 16723417 | 5028447 | *HIPK3* | NM_005734 | 8.52E-05 | 0.767711 | -1.30257 |
| 16652415 | 0 |  | --- | 8.93E-05 | 2.10023 | 2.10023 |
| 16828978 | 5108262 |  | --- | 9.02E-05 | 0.763819 | -1.30921 |
| 16911260 | 5174097 |  | --- | 9.08E-05 | 1.60383 | 1.60383 |
| 16926543 | 5186106 | *POFUT2* | NM_015227 | 9.13E-05 | 3.53874 | 3.53874 |
| 16665191 | 4983182 | *PRKAA2* | NM_006252 | 9.21E-05 | 1.34742 | 1.34742 |
| 17009879 | 5250046 | *PRIM2* | NM_000947 | 9.53E-05 | 2.36213 | 2.36213 |
| 16991141 | 5235851 | *NDST1* | NM_001543 | 9.72E-05 | 0.535644 | -1.86691 |
| 16748459 | 5046078 | *LOH12CR1* | ENST00000543990 | 0.000104126 | 3.2224 | 3.2224 |
| 17103100 | 5320779 | *CDK16* | NM_006201 | 0.000105999 | 1.87844 | 1.87844 |
| 16881667 | 5150930 | *MTHFD2* | ENST00000462026 | 0.000107649 | 0.39415 | -2.53711 |
| 17112704 | 5328107 | *TRMT2B* | NM_024917 | 0.000109346 | 1.3599 | 1.3599 |
| 17048655 | 5278507 | *DYNC1I1* | NM_004411 | 0.000112728 | 0.396061 | -2.52486 |

Table 13. Top transcripts showing alternative splicing between BEN and non-BEN individuals

| **Transcript Cluster ID** | **Gene Symbol** | **RefSeq** | **p-value (BEN)** | **alt-splicing (BEN)** | **# of markers** |
| --- | --- | --- | --- | --- | --- |
| 17113725 | ***LAMP2*** | NM_002294 | 0.309212 | 2.63E-12 | 17 |
| 17076867 | ***PRKDC*** | NM_006904 | 0.921728 | 3.81E-12 | 82 |
| 17068609 | ***FNTA*** | ENST00000302279 | 0.993676 | 2.20E-08 | 21 |
| 16976599 | ***SULT1B1*** | ENST00000310613 | 0.677357 | 6.63E-08 | 13 |
| 16792859 | ***PYGL*** | ENST00000216392 | 0.559887 | 2.77E-07 | 20 |
| 16679033 | ***LGALS8*** | NM_201544 | 0.590633 | 6.50E-07 | 22 |
| 17003858 | ***RNF130*** | ENST00000521389 | 0.7154 | 2.45E-06 | 17 |
| 16765172 | ***SPRYD3*** | NM_032840 | 0.706631 | 2.56E-06 | 15 |
| 16846745 | ***SPAG9*** | NM_001130528 | 0.565004 | 3.34E-06 | 41 |
| 16906749 | ***HECW2*** | ENST00000260983 | 0.37147 | 6.55E-06 | 31 |
| 16810585 | ***PIF1*** | NM_025049 | 0.699193 | 7.47E-06 | 17 |
| 16886503 | ***RIF1*** | NM_018151 | 0.312811 | 9.84E-06 | 41 |
| 17084523 | ***IL11RA*** | NM_001142784 | 0.0155357 | 1.05E-05 | 19 |
| 16671791 | ***MSTO1*** | NR_046294 | 0.535131 | 1.34E-05 | 8 |
| 16822383 | ***RGS11*** | NM_183337 | 0.401345 | 1.42E-05 | 21 |
| 16940233 | ***TMIE*** | NM_147196 | 0.0127063 | 2.01E-05 | 5 |
| 16953052 | *SETD2* | NM_014159 | 0.78481 | 3.28E-05 | 26 |
| 17095138 | *OTTHUMG00000020070* | OTTHUMT00000052782 | 0.407224 | 3.72E-05 | 5 |
| 17012767 | *TBPL1* | NM_001253676 | 0.346497 | 4.60E-05 | 12 |
| 16972643 | *LOC285501* | NR_028342 | 0.893765 | 4.92E-05 | 7 |
| 16751993 | *HNRNPA1* | NM_031157 | 0.436966 | 5.49E-05 | 15 |
| 16940325 | *NRADDP* | NR_024046 | 0.0510897 | 5.78E-05 | 3 |
| 16810686 | *CLPX* | ENST00000300107 | 0.62304 | 6.85E-05 | 21 |
| 16847095 | *RNF43* | NM_017763 | 0.160878 | 8.00E-05 | 11 |
| 16752368 | *IKZF4* | NM_022465 | 0.69805 | 8.24E-05 | 18 |
| 16993538 | *BTNL8* | NM_024850 | 0.567595 | 8.34E-05 | 14 |
| 16934660 | *TEX33* | NM_001163857 | 0.131045 | 8.48E-05 | 10 |
| 16996956 | *SMA4* | NR_024054 | 0.32729 | 8.88E-05 | 4 |
| 16700274 | *ACTA1* | NM_001100 | 0.825752 | 8.90E-05 | 5 |
| 16882375 | *PTCD3* | NM_017952 | 0.428754 | 8.94E-05 | 34 |
| 16768438 | *BTG1* | NM_001731 | 0.112882 | 9.54E-05 | 21 |
| 17021922 | *PNISR* | ENST00000369239 | 0.354934 | 9.82E-05 | 23 |

**Bold** FDR < 0.05

Table 14. Transcripts that are alternatively spliced but not differentially expressed between BEN and non-BEN individuals

| **Transcript Cluster ID** | **Gene symbol** | **Refseq** | **P (alt splice)** | **P (BEN vs non-BEN)** |
| --- | --- | --- | --- | --- |
| 17068609 | *FNTA* | ENST00000302279 | 2.20E-08 | 0.993676 |
| 16749583 | *FAR2* | NM_001271783 | 0.000294308 | 0.971996 |
| 16872166 | *FBXO27* | NM_178820 | 0.00113119 | 0.965235 |
| 17024508 | *STXBP5-AS1* | NR_034115 | 0.00123378 | 0.993366 |
| 16779975 | *LINC00439* | OTTHUMT00000045354 | 0.00261979 | 0.969214 |
| 16777401 | *MIPEP* | NM_005932 | 0.00284841 | 0.96252 |
| 17085599 | *OTTHUMG00000019960* | OTTHUMT00000052540 | 0.00306952 | 0.972756 |
| 17109487 | *RS1* | ENST00000379984 | 0.00601389 | 0.992496 |
| 16859205 | *UCA1* | NR_015379 | 0.00608228 | 0.968412 |
| 16685729 | *BMP8B* | ENST00000372827 | 0.00637766 | 0.99282 |
| 16680446 | *NADK* | NM_001198994 | 0.00647953 | 0.952229 |
| 16940154 | *CXCR6* | ENST00000304552 | 0.00656796 | 0.985194 |
| 16686510 | *GPBP1L1* | NM_021639 | 0.00856819 | 0.96696 |
| 16778166 | *ALG5* | NM_013338 | 0.00900682 | 0.993358 |
| 16669255 | *MAN1A2* | ENST00000356554 | 0.00932624 | 0.986574 |
| 16698883 | *LAMB3* | NM_001017402 | 0.0108313 | 0.984948 |
| 16942659 | *PPP4R2* | ENST00000295862 | 0.0111172 | 0.950559 |
| 16915771 | *COL9A3* | NM_001853 | 0.0112645 | 0.991523 |
| 17052115 | *TRIM24* | NM_015905 | 0.011682 | 0.994098 |
| 16943763 | *PVRL3* | NM_001243288 | 0.0118664 | 0.968679 |
| 16780423 | *OXGR1* | NM_080818 | 0.0141236 | 0.961597 |
| 17093620 | *PIGO* | NM_032634 | 0.0143667 | 0.967159 |
| 16924192 | *LIPI* | ENST00000344577 | 0.0155174 | 0.974185 |
| 16829422 | *CENPBD1* | NM_145039 | 0.0171109 | 0.999063 |
| 16750597 | *C12orf54* | ENST00000314014 | 0.0179514 | 0.977917 |
| 16971406 | *LOC729558* | AK096995 | 0.0189211 | 0.951726 |
| 16677748 | *MIA3* | NM_198551 | 0.0206515 | 0.999924 |
| 16777460 | *PARP4* | NM_006437 | 0.0210708 | 0.961285 |
| 17016043 | *MBOAT1* | NM_001080480 | 0.0212037 | 0.974343 |
| 16690816 | *PGCP1* | NR_029429 | 0.021219 | 0.95585 |
| 16849142 | *FOXJ1* | NM_001454 | 0.0221304 | 0.972029 |
| 17067890 | *ZNF703* | ENST00000331569 | 0.0227231 | 0.991107 |
| 17116177 | *OTTHUMG00000041597* | OTTHUMT00000099586 | 0.0241531 | 0.981746 |
| 17017053 | *CDSN* | NM_001264 | 0.026121 | 0.974013 |
| 16697492 | *KCNT2* | NM_198503 | 0.0268824 | 0.994306 |
| 17047514 | *ZP3* | ENST00000394857 | 0.0274385 | 0.992742 |
| 16670894 | *TUFT1* | NM_020127 | 0.0275081 | 0.968354 |
| 16767229 | *OTTHUMG00000169088* | OTTHUMT00000402215 | 0.0275457 | 0.976058 |
| 16707534 | *CYP26C1* | NM_183374 | 0.0289495 | 0.989232 |
| 17049003 | *LOC100289187* | NM_001195541 | 0.0298453 | 0.984599 |
| 16960106 | *OTTHUMG00000159376* | OTTHUMT00000355002 | 0.0311542 | 0.992506 |
| 16710073 | *BTBD16* | NM_144587 | 0.031908 | 0.954581 |
| 16962628 | *TPRG1-AS2* | NR_046722 | 0.032553 | 0.955494 |
| 16724269 | *CREB3L1* | NM_052854 | 0.0327585 | 0.972572 |
| 17071640 | *CTHRC1* | NM_138455 | 0.0364357 | 0.987081 |
| 16885432 | *UGGT1* | NR_027671 | 0.0365169 | 0.997055 |
| 16747402 | *LAG3* | NM_002286 | 0.0384148 | 0.969989 |
| 17107309 | *VGLL1* | NM_016267 | 0.0400684 | 0.957934 |
| 16662676 | *C1orf122* | NM_198446 | 0.0414201 | 0.952767 |
| 16858729 | *MAST1* | NM_014975 | 0.0421134 | 0.987321 |
| 17054748 | *ZNF890P* | NR_034163 | 0.0423093 | 0.960867 |
| 16867915 | *INSR* | NM_000208 | 0.0436146 | 0.953119 |
| 16878069 | *EMILIN1* | NM_007046 | 0.0462787 | 0.977786 |
| 16715626 | *OTTHUMG00000176185* | OTTHUMT00000431608 | 0.0473977 | 0.97584 |
| 16821229 | *OTTHUMG00000176979* | OTTHUMT00000434558 | 0.0477629 | 0.986008 |
| 17101554 | *LOC100093698* | NR_046087 | 0.0487691 | 0.966227 |

Table 15. Association of rs2814778 *CC* recessive genotype with cytokines in African-American cohorts

| Cytokine | Cohort | N | β(SE) | p |
| --- | --- | --- | --- | --- |
| CRP | HUFS | 1623 | 0.099 (0.026) | <0.001* |
|  | MESA | 1344 | 0.148 (0.061) | 0.015* |
| IL-6 | HUFS | 825 | 0.031 (0.023) | 0.190 |
|  | MESA | 1306 | 0.018 (0.034) | 0.600 |
| IL-10 | HUFS | 828 | 0.002 (0.009) | 0.820 |
| Il-2 | MESA | 649 | 0.014 (0.030) | 0.655 |
| MMP-9 | MESA | 169 | -0.439 (0.084) | <0.001* |
| MMP-3 | MESA | 169 | 0.065 (0.075) | 0.388 |

Association with log-transformed cytokine values under a recessive model adjusting for age, sex, body mass index and type 2 diabetes

**Supporting Figures**

Figure 1. Principal component (PC) plots of the genotypes in the discovery (REGARDS) sample


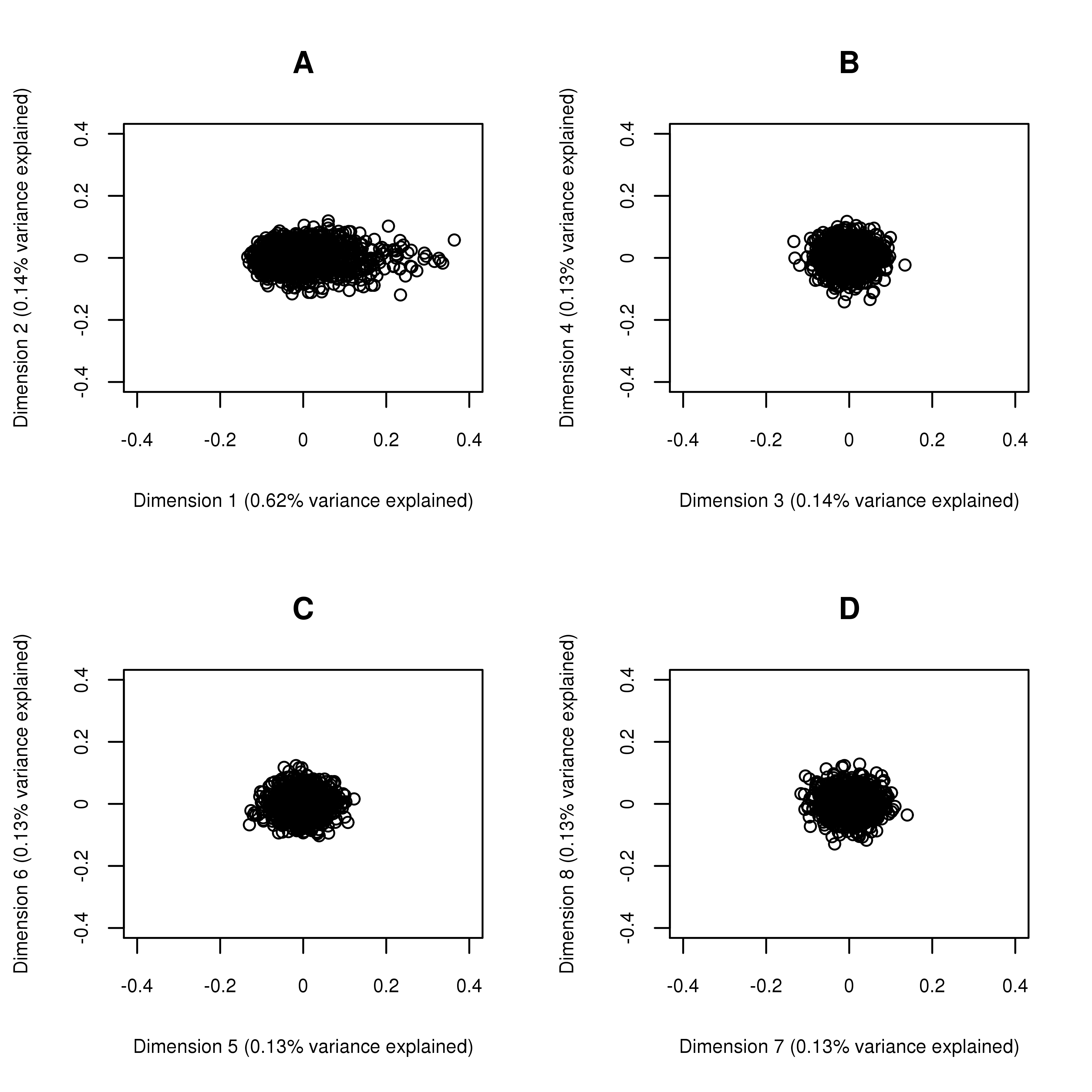


Figure 2. PC plots of discovery (REGARDS) sample with International HapMap Project reference populations


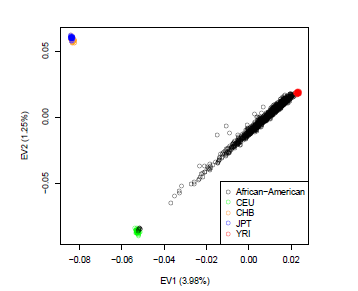


CEU = CEPH European ancestry from Utah; CHB = Han Chinese in Beijin; JPT = Japanese in Tokyo; YRI Yourba in Ibadan, Nigeria

Figure 3. Scree plot of the discovery (REGARDS) sample


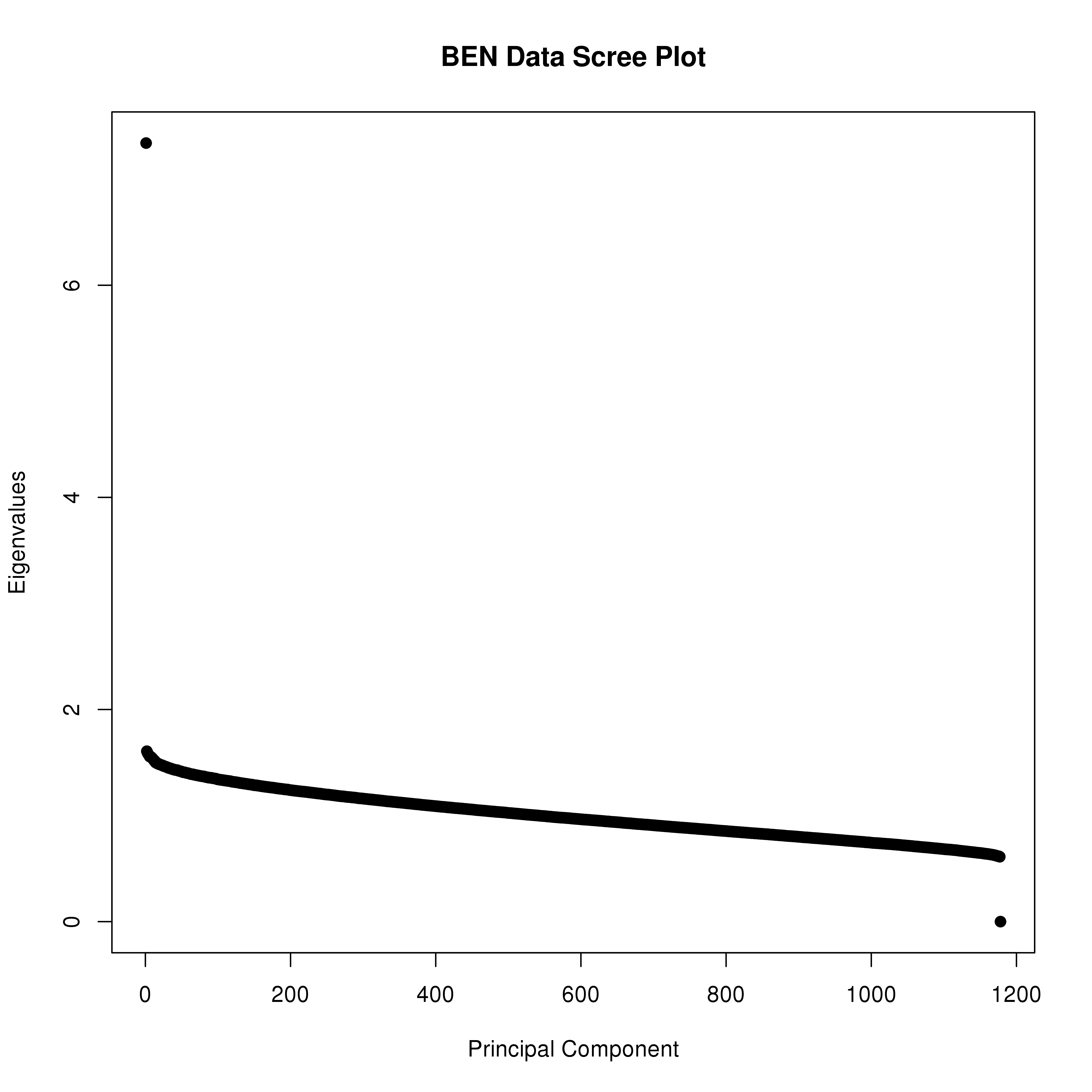


Figure 4. Plot of selection around the *DARC* locus on chromosome 1 across 1 MB (upper panel) and zoomed in to 500 kb (lower panel)

| 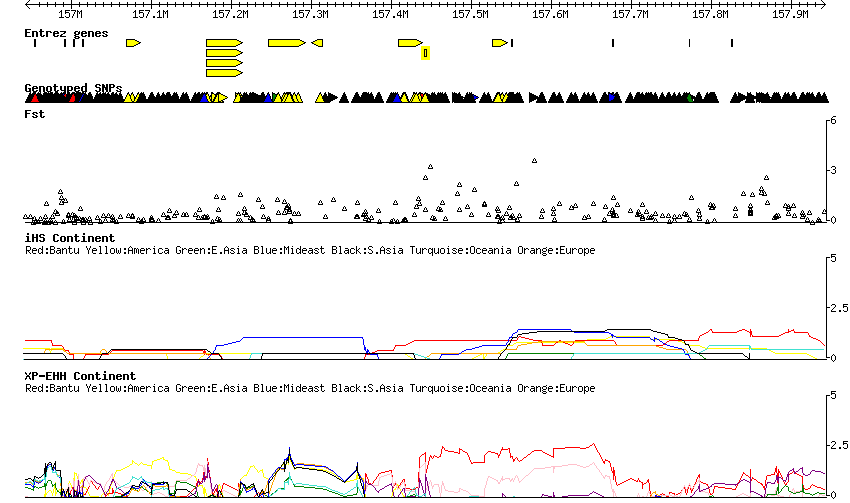 |
| --- |
| 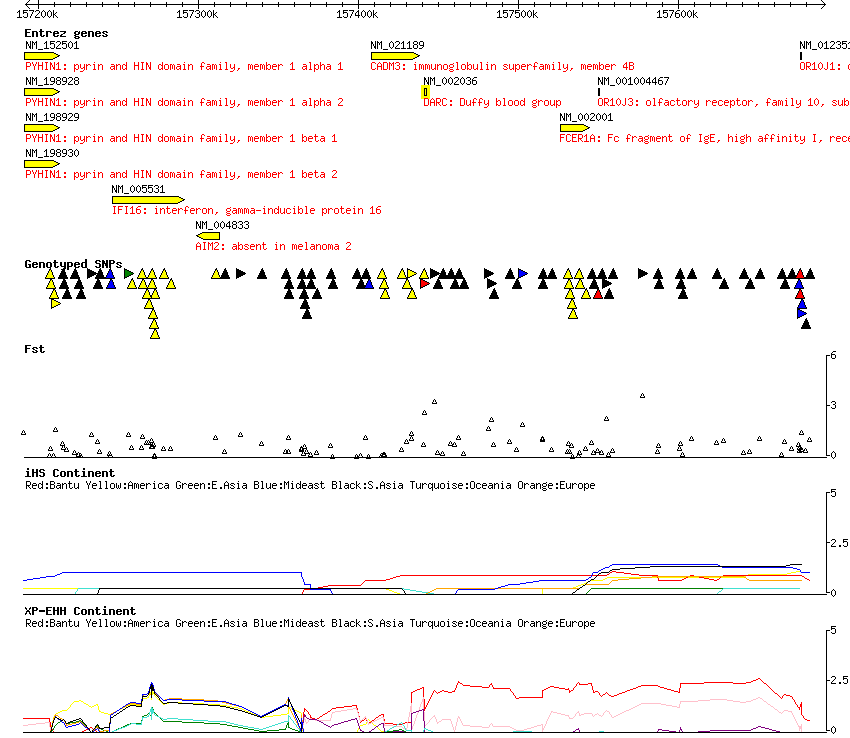 |

Figure 5. QQ plot of association statistics in the discovery (REGARDS) sample with (a) all markers (b) exclusion of chromosome 1 region

(a)


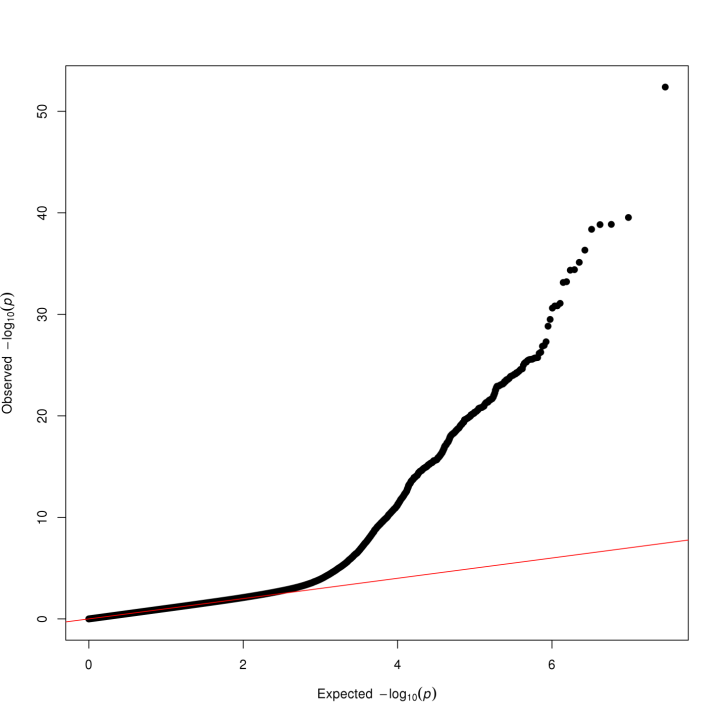


(b)

Figure 6. Manhattan plot of the discovery (REGARDS) sample: (a) genotyped SNPs only, (b) genotyped + imputed SNPs

(a)


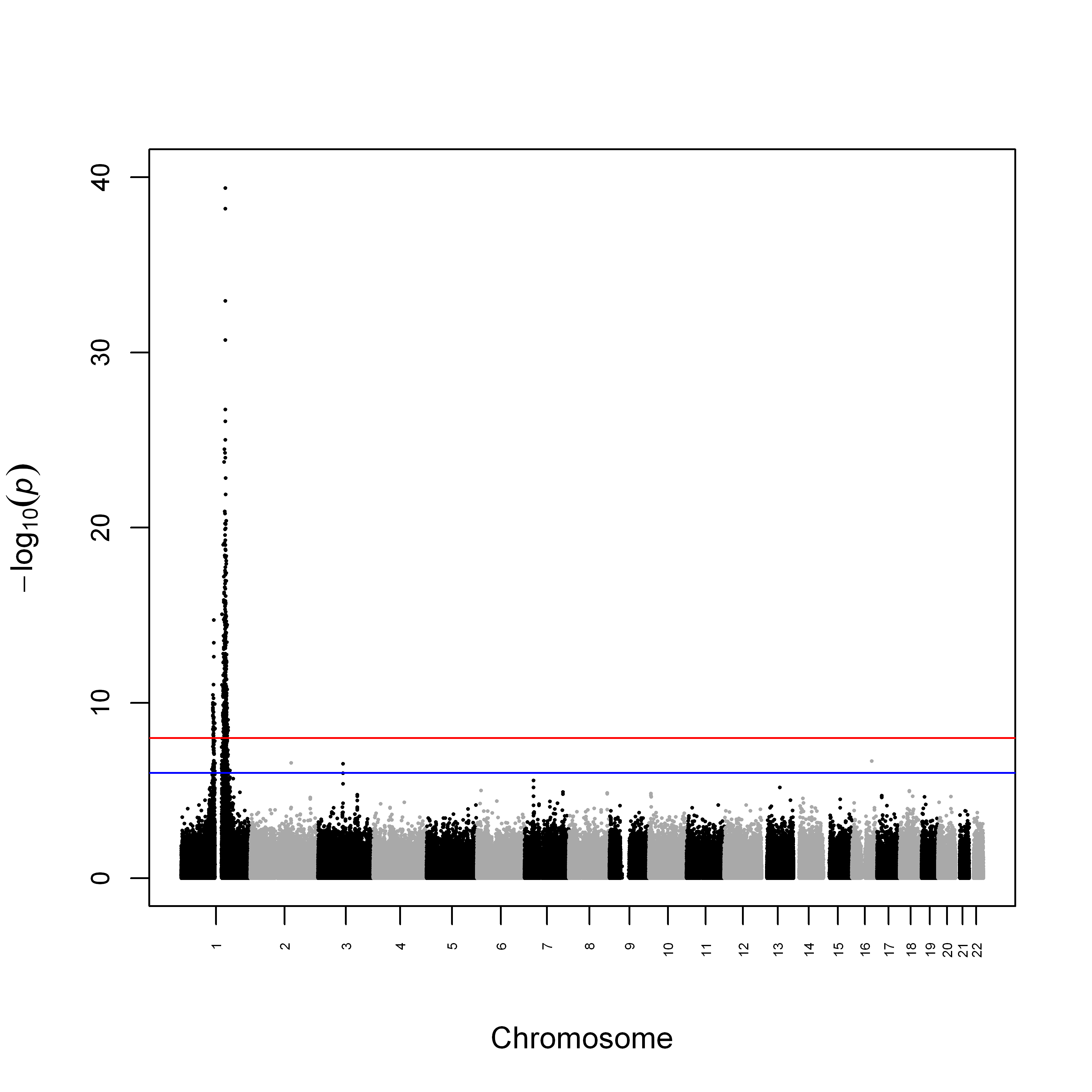


(b)


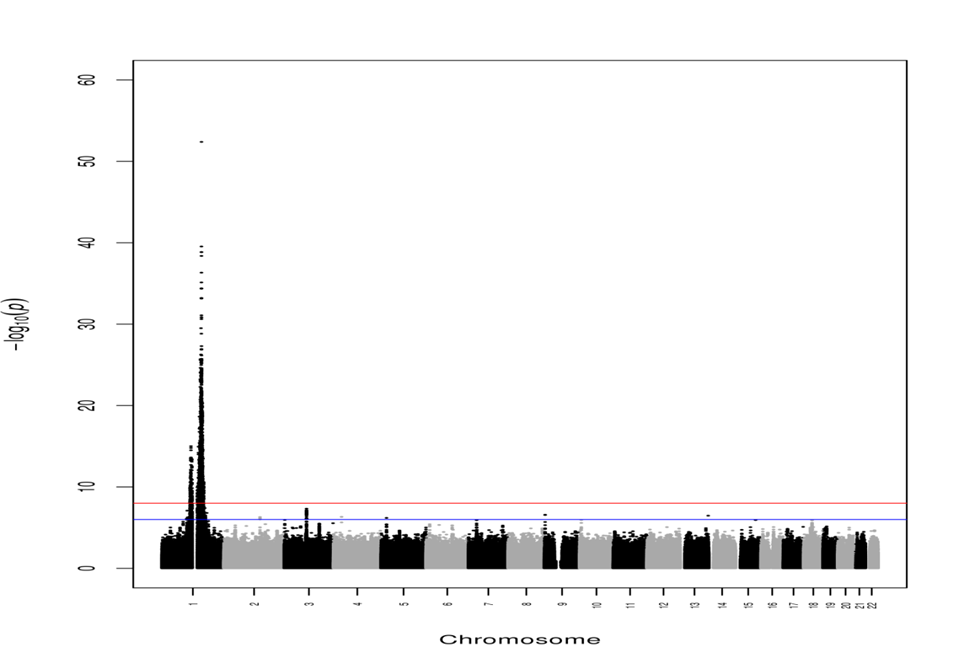


Figure 7. Manhattan plot of the discovery (REGARDS) sample conditioning on leading *DARC* SNP rs2814778


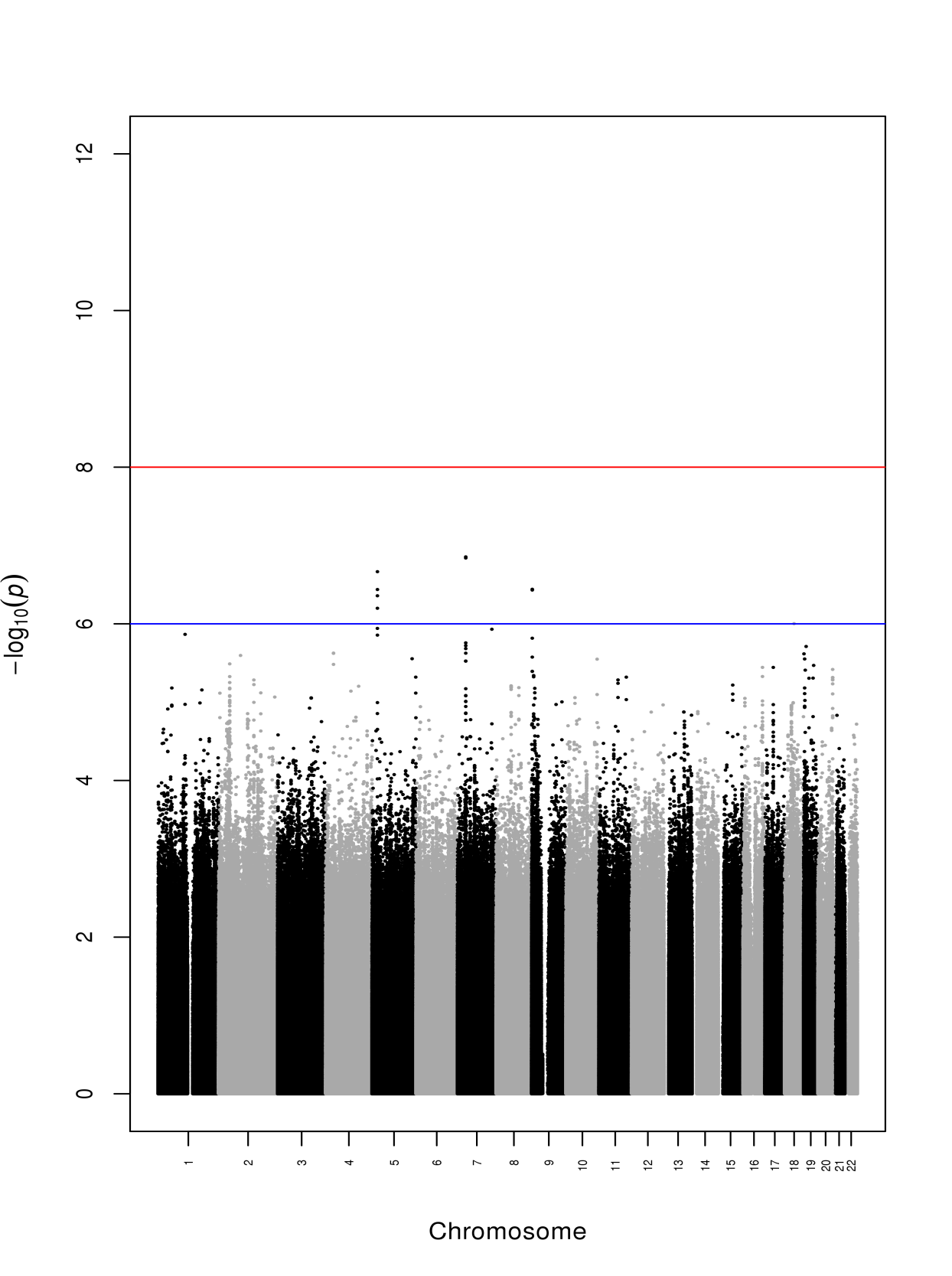


Figure 8. Principal component (PC) plots of the genotypes in the replication (ARIC) sample


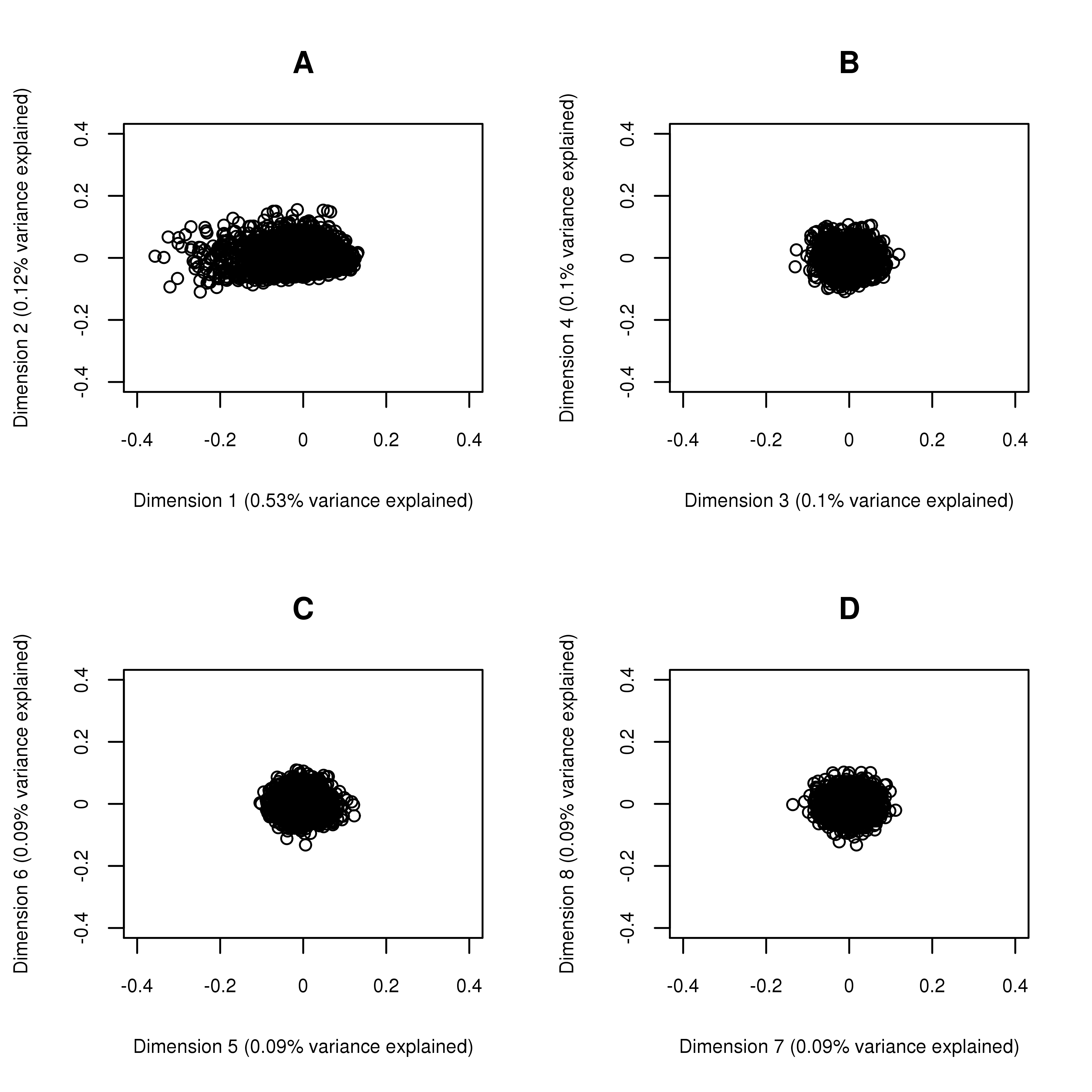


Figure 9. Scree plot of the replication (ARIC) sample


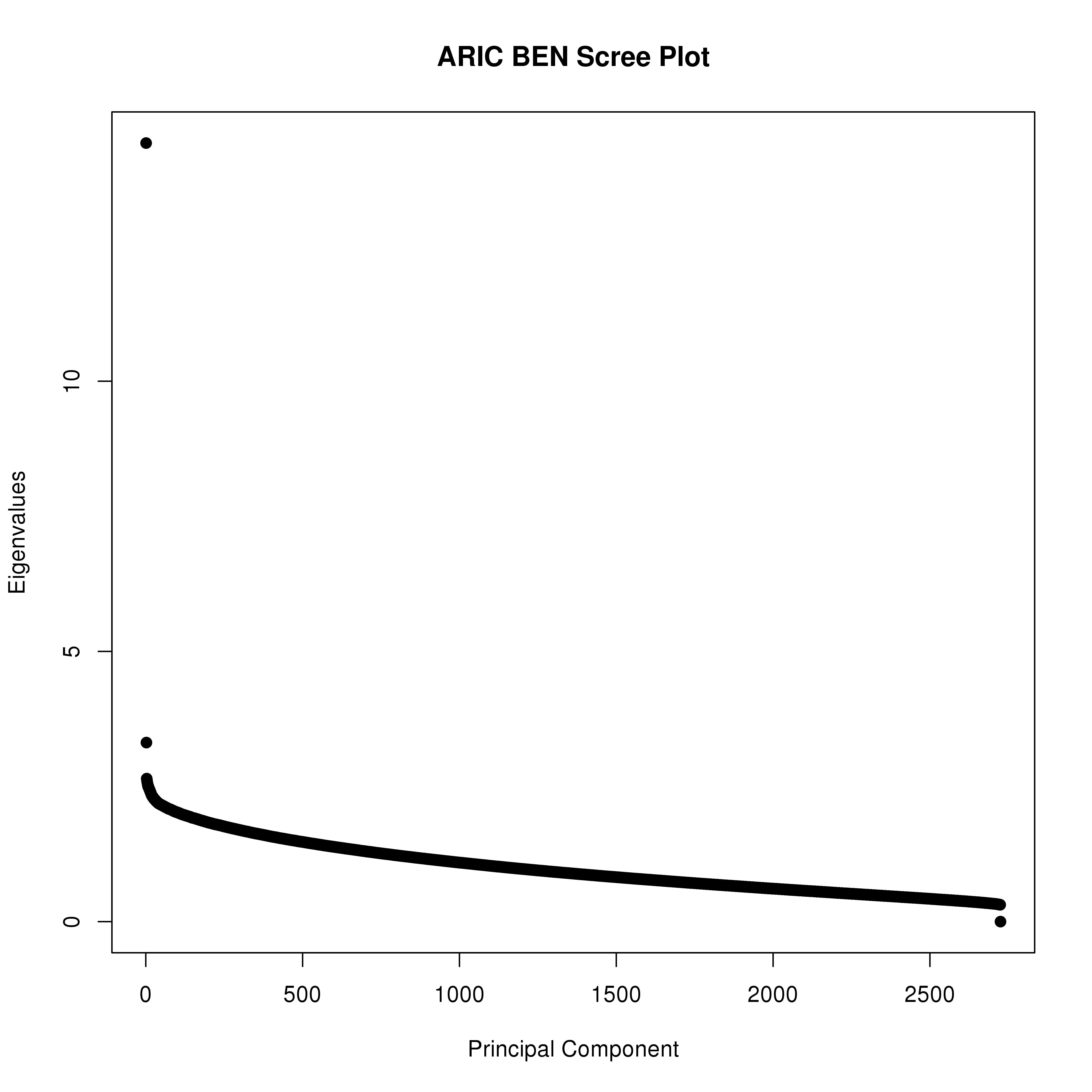


Figure 10. Manhattan plot of the replication (ARIC) sample


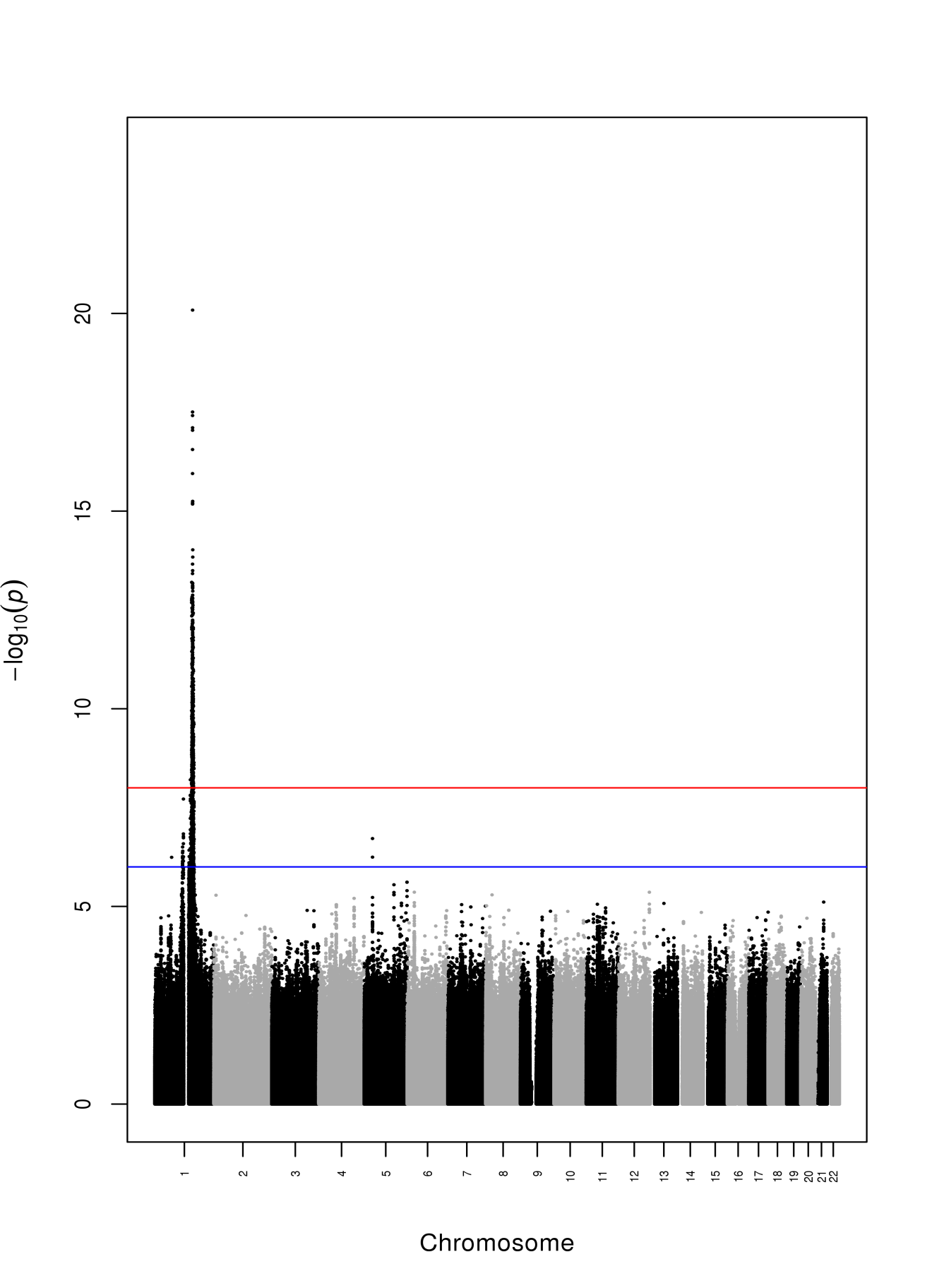


Figure 11. Manhattan plot of the replication (ARIC) sample conditioning on leading *DARC* SNP rs2814778


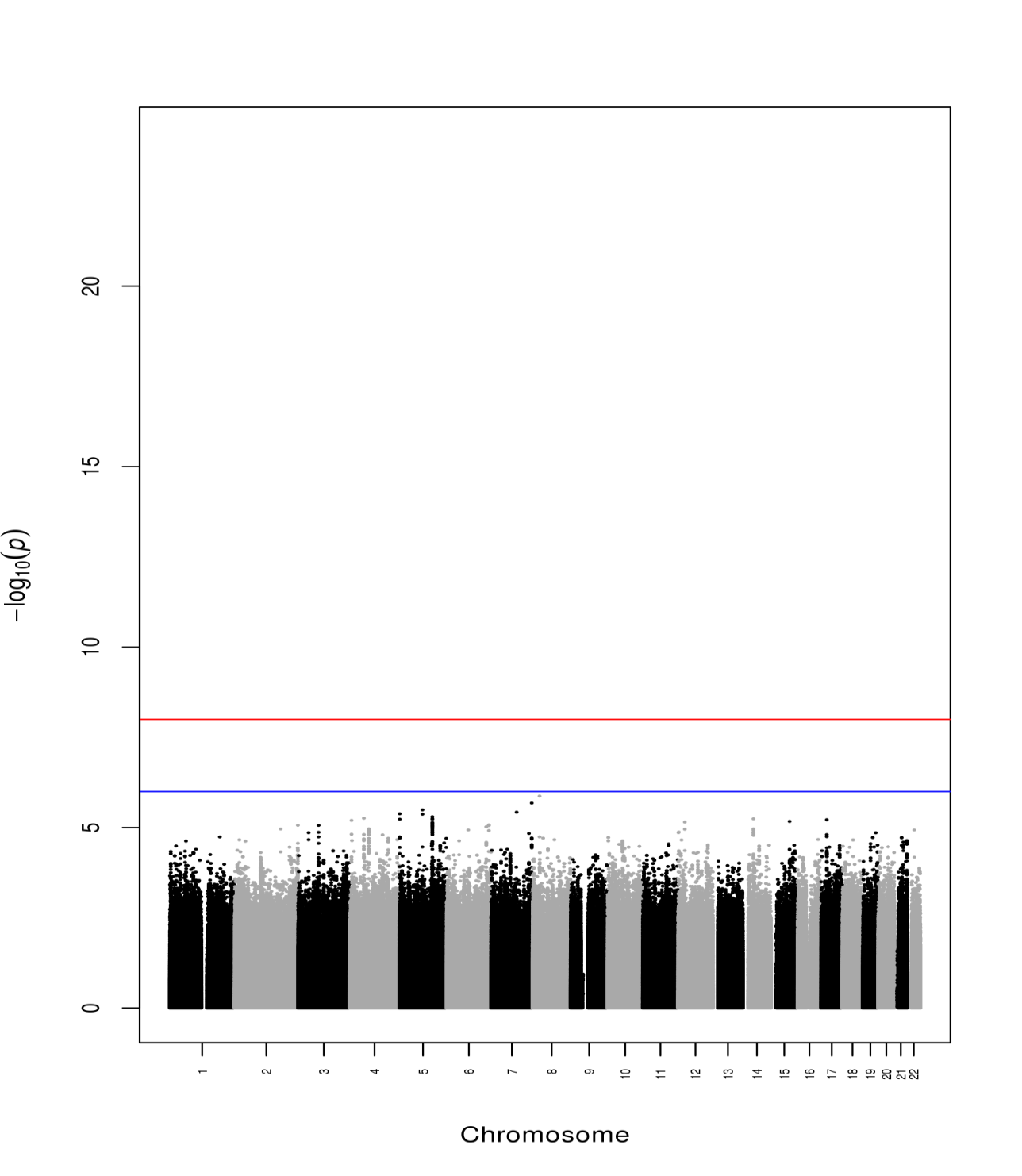


Figure 12. Quality control figures for gene expression study

| A  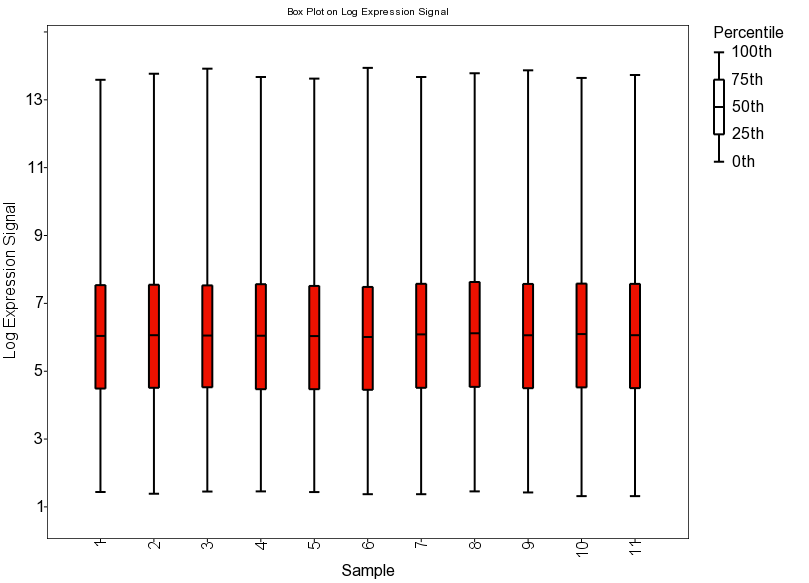 | B  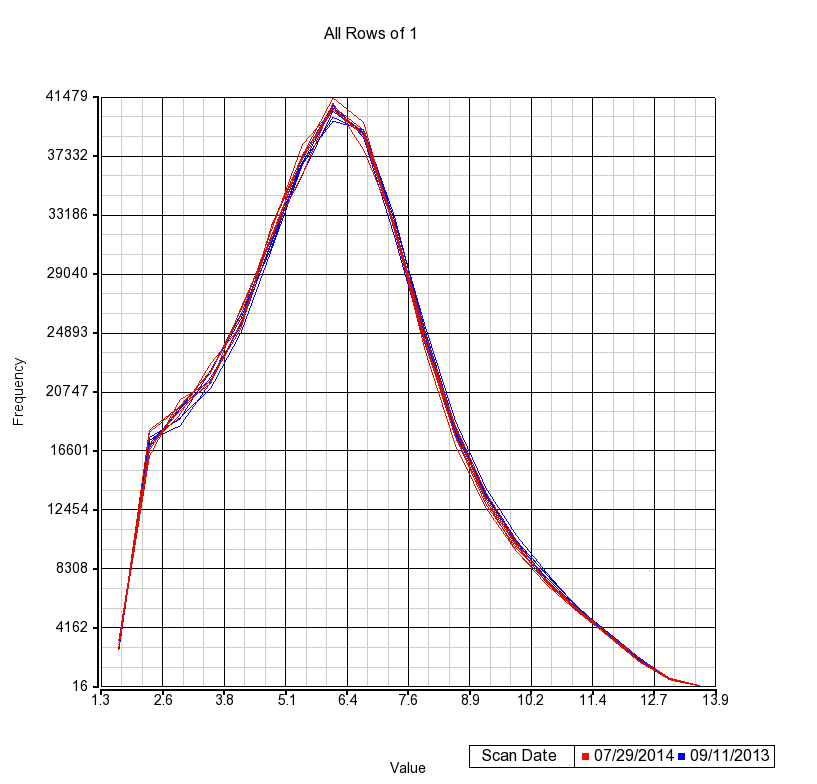 |
| --- | --- |
| C  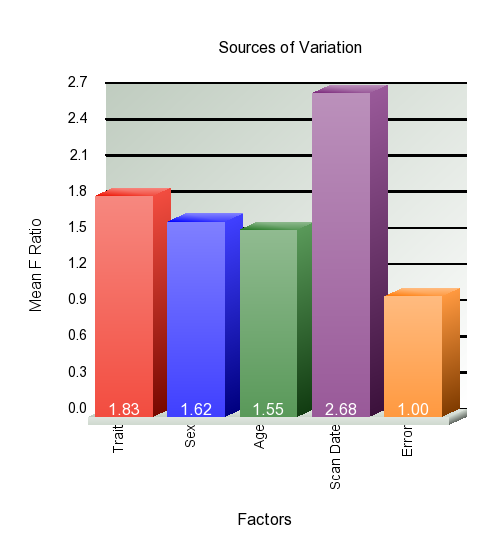 | D  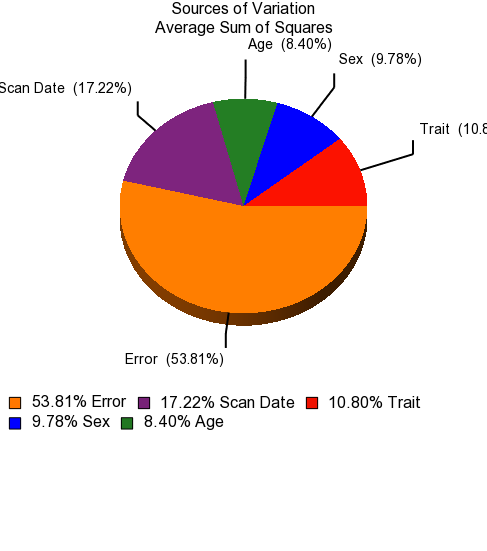 |

Figure 13. Unsupervised hierarchical clustering heat map of probe sets of genes (full-length transcripts) with p <5 X 10^-4^ for differential expression between BEN and non-BEN individuals

| A  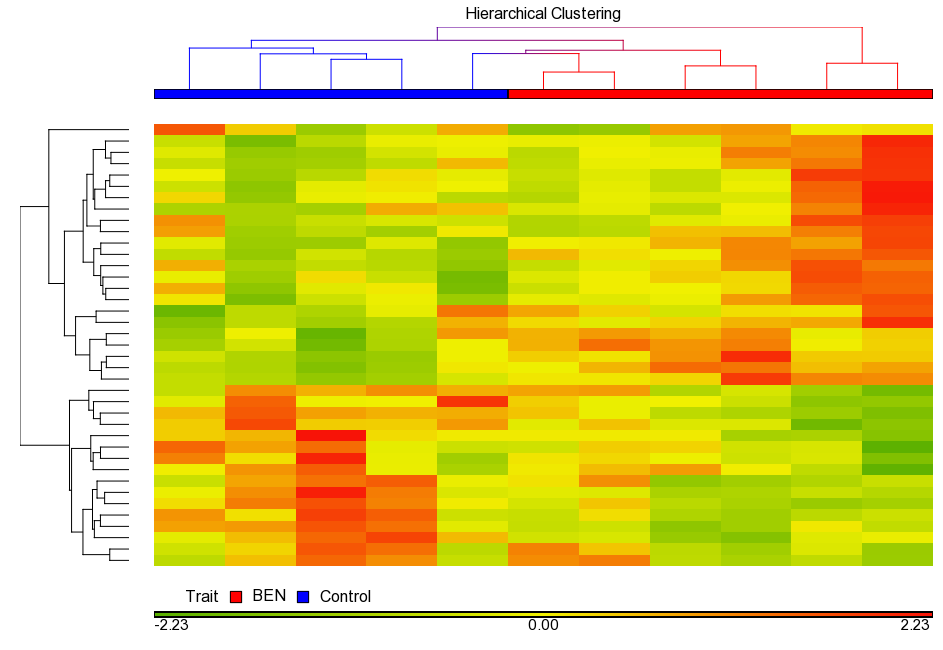 |
| --- |
| B  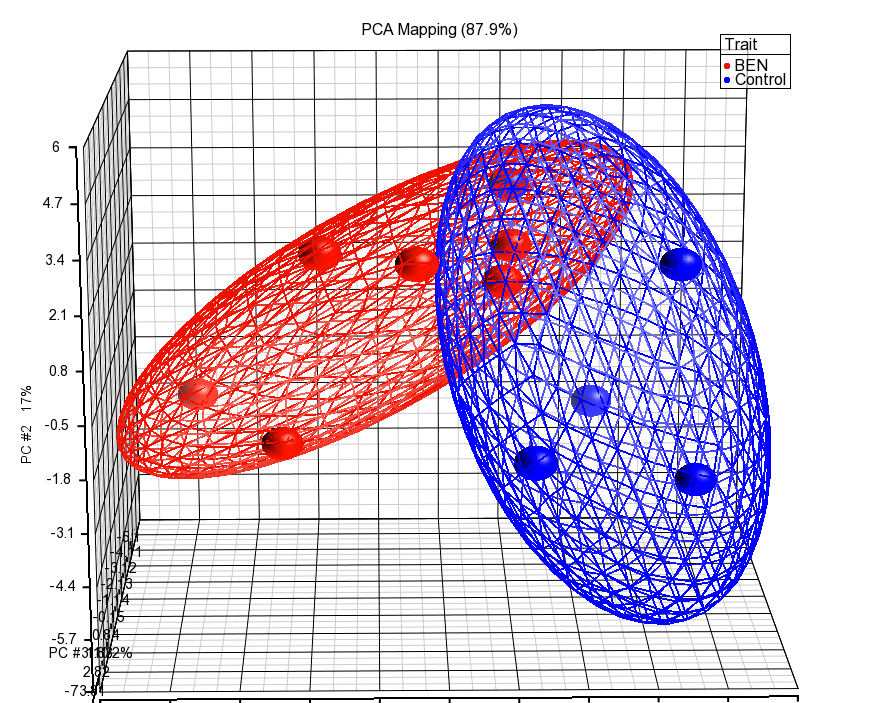 |

Figure 14. Unsupervised hierarchical clustering heat map of probe sets of exons with p <1 X 10^-4^ for differential expression between BEN and non-BEN individuals

| A  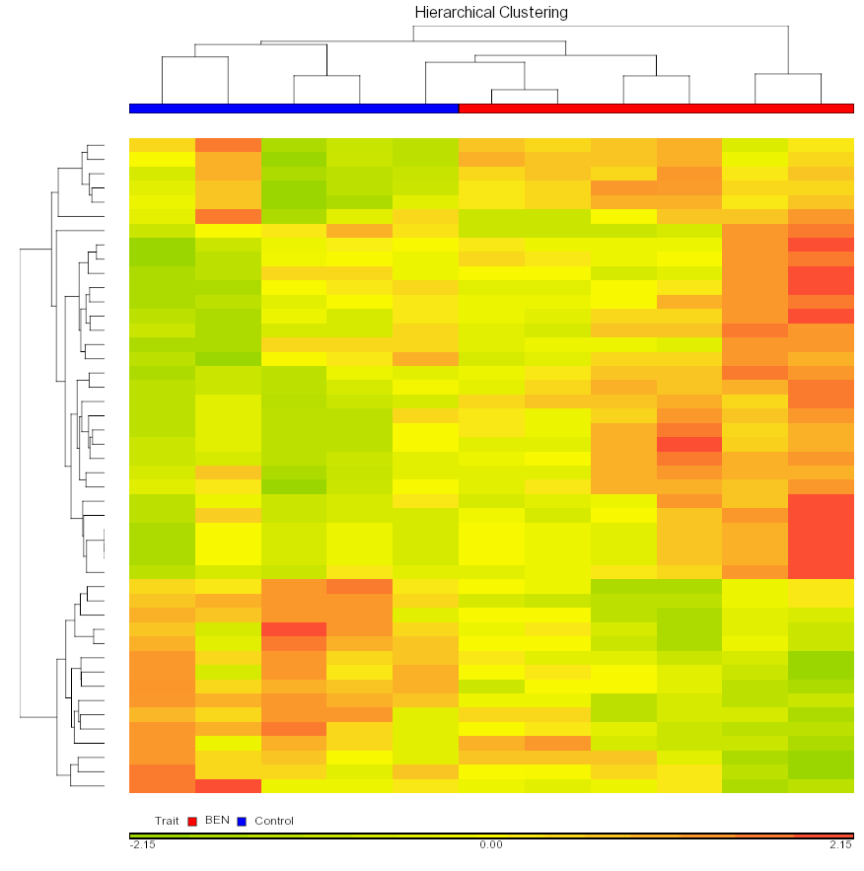 |
| --- |
| B  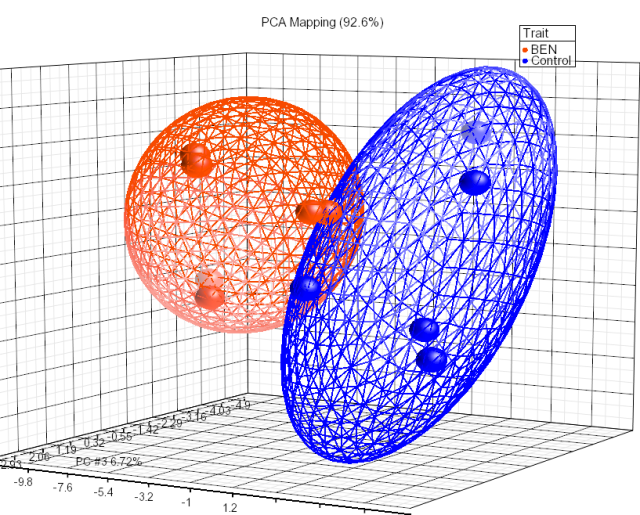 |

Figure 15. Pathway Maps enriched in set of differentially expressed transcripts in (a) hematologic/immunologic tissues, (b) all tissues

(a)


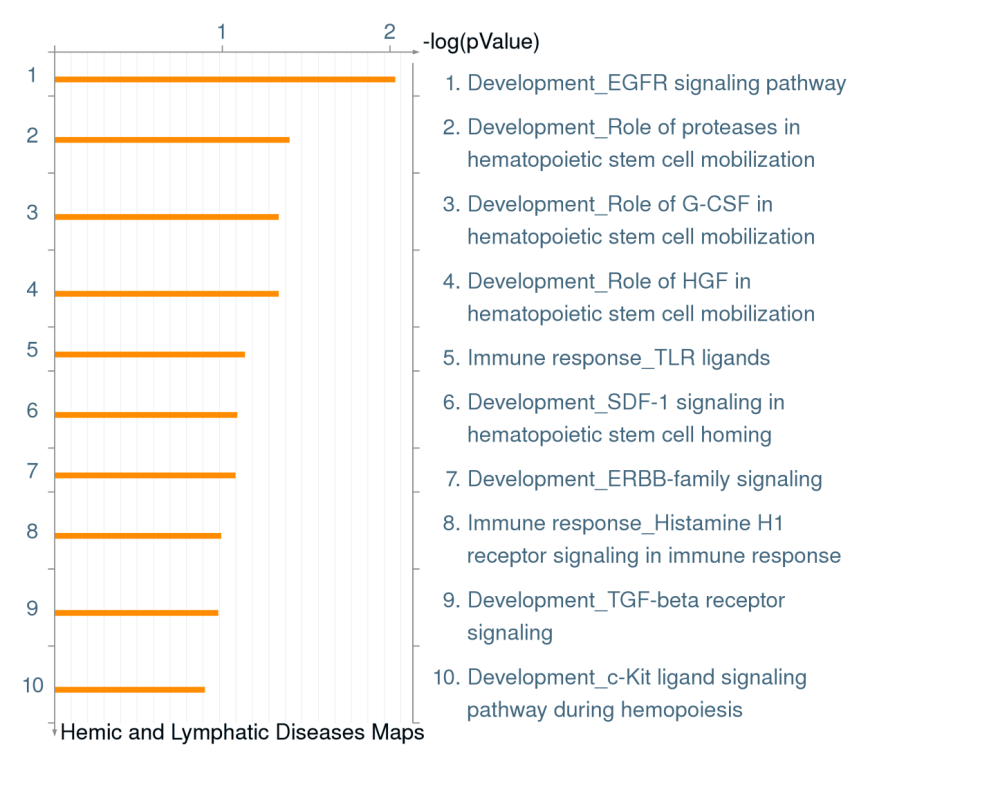


(b)


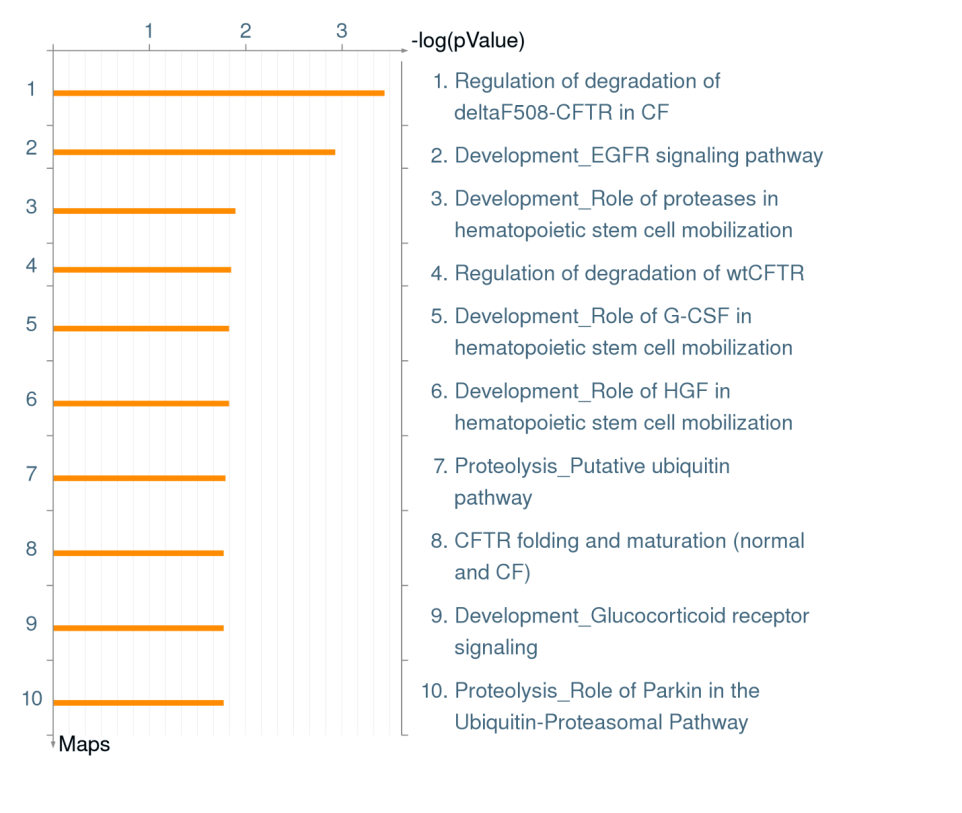


Figure 16. Gene ontology (GO) enrichment scores of biological processes in hematologic/immunologic tissues of differentially expressed transcripts


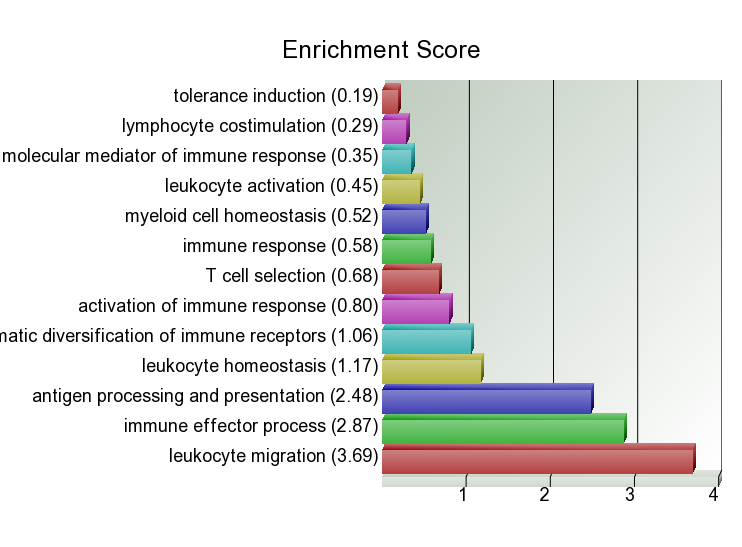


Figure 17. *DARC* (*ACKR1*) expression in multiple tissues including several white cell lineages

(source: BioGPS - <http://biogps.org/#goto=genereport&id=2532>, accessed 11/24/2014)


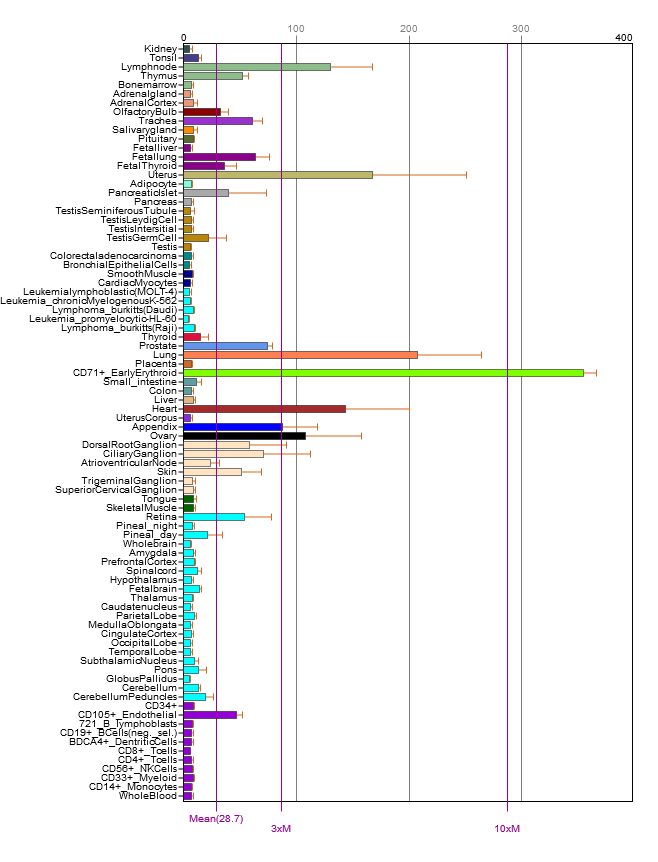


Figure 18. *DARC* (*ACKR1*) expression profiles in selected GEO datasets (*Homo sapiens* only)

| 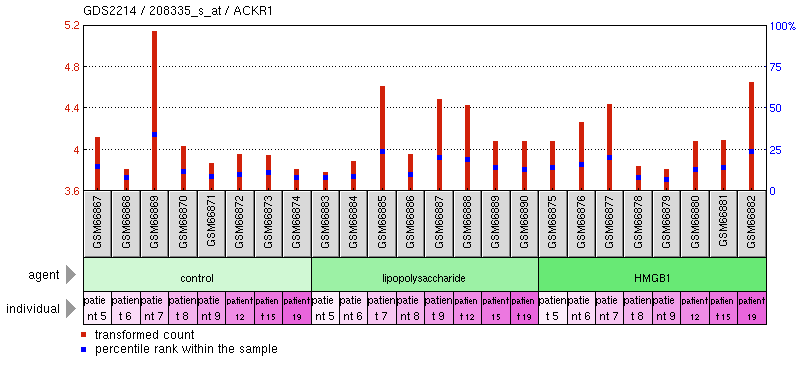 |
| --- |
| 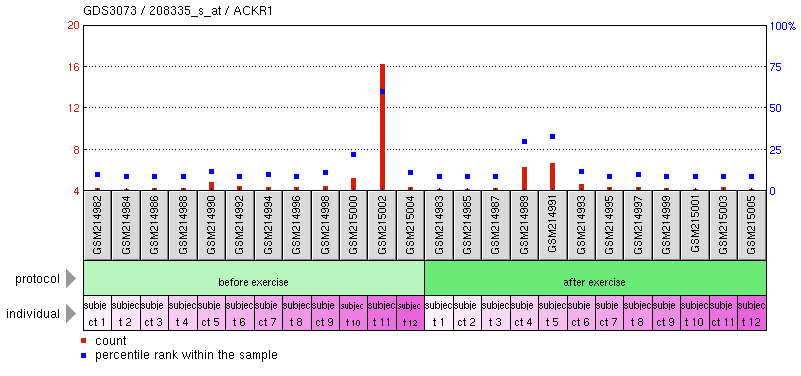 |
| 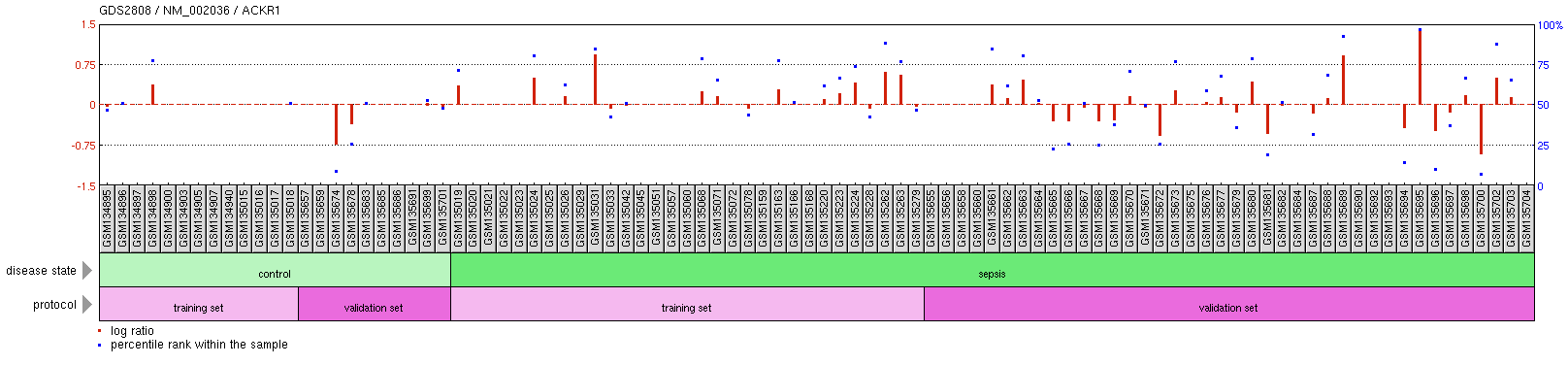 |
| 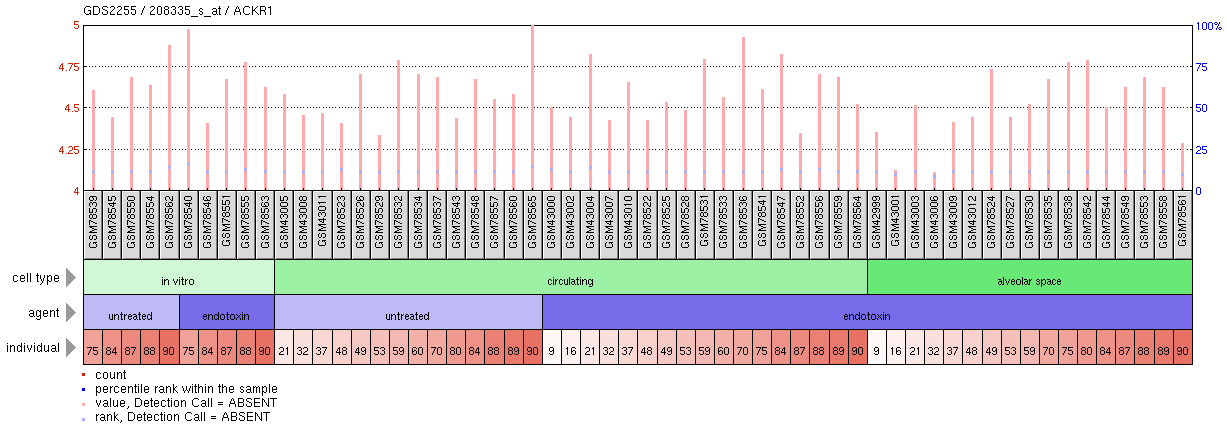 |
